# Utilization of wastes from bioethanol production for the fabrication of new adsorbents for the removal of toxic dye in water

**DOI:** 10.1038/s41598-026-35236-8

**Published:** 2026-01-27

**Authors:** Khloud Eltaher, Sara E. AbdElhafez, Rehab M. Ali, Ayman El-Faham, Ali A. El-Bardan, Hesham Hamad

**Affiliations:** 1 Department of Chemistry, Faculty of Science, Alexandria University, P.O. Box 426, Ibrahimia, 21321 Alexandria Egypt; 2 Fabrication Technology Research Department, Advanced Technology and New Materials Research Institute (ATNMRI), City of Scientific Research and Technological Applications (SRTA-City), New Borg El-Arab City 21934, Alexandria, Egypt

**Keywords:** Adsorption, Lignosulfonates, Cationic dyes, Adsorption mechanisms, SDGs, Environmental chemistry, Chemistry, Materials chemistry

## Abstract

Cationic dyes, even at low concentrations, pose serious risks to aquatic ecosystems and contribute to environmental pollution. Effective removal of these dyes is therefore essential for protecting water resources and marine life. This study investigates lignosulfonate-based adsorbents (L, LS, and LSR-F) for the removal of crystal violet (CV) dye from wastewater. Structural and physicochemical characterization using FTIR, Raman spectroscopy, SEM, TGA, and CHNS analysis confirmed the successful synthesis and functionalization of the adsorbents. Under optimal conditions (pH 8, 150 mg/L CV, 0.1 g adsorbent dose, room temperature), LSR-F achieved 98% removal efficiency within 15 min. Kinetic modeling with five approaches (pseudo-first-order, pseudo-second-order, intraparticle diffusion, Boyd, and Elovich) revealed that adsorption followed the pseudo-second-order model, suggesting chemisorption as the dominant mechanism. Isotherm studies indicated that the Langmuir model best described the adsorption process, with LSR-F exhibiting a maximum adsorption capacity of 73.53 mg/g. Thermodynamic analysis further showed that the process was spontaneous and endothermic. Overall, LSR-F demonstrates excellent potential as a cost-effective, sustainable adsorbent for removing toxic cationic dyes from wastewater. By utilizing bio-industrial residues, this approach supports multiple Sustainable Development Goals (SDGs), particularly SDG 6 (Clean Water and Sanitation), SDG 12 (Responsible Consumption and Production), SDG 13 (Climate Action), and SDG 14 (Life Below Water).

**Supplementary Information:**

The online version contains supplementary material available at 10.1038/s41598-026-35236-8.

## Introduction

 The rapid growth of industrial activities in recent decades has led to significant environmental pollution caused by hazardous organic and inorganic substances. Dyes, particularly colored ionized aromatic organic pollutants, are commonly used in various industries such as textiles, paint, printing, food, paper, leather, cosmetics, and plastics^1^. These dyes have detrimental effects on human health, including poisoning, carcinogenicity, and mutagenicity^2^. Furthermore, the effluents containing these dyes often consist of cationic, anionic, and azo dyes. Among the various dyes used in industries, crystal violet (CV) is of particular concern. CV, a cationic triphenylmethane dye, is employed as a biological stain, textile colorant, and paper dye. Exposure to CV can lead to respiratory and renal failure, skin and digestive tract irritation, and mammalian cell toxicity^3^. Consequently, the decolorization and treatment of dyed wastewater have become imperative.

Various technologies have been investigated for removing pollutants from wastewater, including reverse osmosis, membrane filtration, chemical precipitation, solvent extraction, biological treatment, oxidation, chemical coagulation/flocculation, irradiation, ion exchange, ozonation, and adsorption. Among the available methods, adsorption is regarded as particularly promising due to its high efficiency, broad availability, operational simplicity, environmental safety, and overall accessibility. Adsorption typically occurs on the pore walls of porous solids, making activated carbon, activated alumina, silica gel, molecular sieves, synthetic resins, zeolites, and chitosan composites common adsorbents^3^.

Agricultural waste generally has low economic value and poses disposal challenges. Annually, around 4 billion tonnes of lignocellulosic fiber byproducts are produced, with 60% from agriculture and 40% from forests^4^. Many studies have investigated these byproducts as adsorbents. These lignocellulosic materials can be used raw or activated to remove CV dye from wastewater. Bioethanol is a renewable and sustainable source of energy that has gained significant attention as an alternative to fossil fuels. The substitution of petrol ethanol with bioethanol offers significant potential for reducing CO_2_ emissions^5^. It is produced through the fermentation of plant-based feedstocks, such as sugarcane, rice straw, corn stover, and cellulosic materials. Utilizing agricultural waste serves several purposes: it mitigates environmental issues by converting surplus waste that is often burned into valuable adsorbents, reduces costs compared to traditional adsorbent production, and emerges as a promising solution for reducing greenhouse gas emissions and mitigating climate change.

Corn stover consists primarily of lignin, cellulose, hemicellulose, and trace amounts of ash. Moreover, it has abundant sources, low cost, consistent chemical and physical properties, wide applicability, and high recyclability. To produce corn stover biomass suitable for bioethanol production, a pretreatment process is employed. This process disrupts the lignocellulosic complex by removing lignin^6^. The resulting lignin byproduct is a carbon-rich solid material that has potential applications after modifications as a valuable resource in various industries. It exhibits excellent adsorption capacities for hydrophobic organic pollutants due to its aromaticity and surface functionalities^7^. Lignin by-product is further modified through surface activation with sodium thiosulfate (Na_2_SO_3_).

Lignin removal and valorization remain central challenges in lignocellulosic biomass pretreatment for bioethanol production. During processes involving mild organic acids, metal salts, and combined thermal treatments, a significant fraction of lignin is separated to facilitate enzymatic saccharification and increase glucose yield. For example, oxalic acid combined with autoclaving has been reported as a green pretreatment strategy that enhances delignification compared to ultrasound irradiation, thereby improving cellulose accessibility and subsequent ethanol yield^8^. Likewise, ZnCl₂ and Na₂HPO₄–ZnCl₂ (NZ) pretreatments under autoclaving have shown considerable structural modifications of corn stover (CS) and Tetra Pak (TP) wastes, with physicochemical analyses confirming efficient lignin disruption, which translated into improved enzymatic hydrolysis and higher ethanol production^9^. Beyond enabling sugar release, these pretreatments generate lignin-rich fractions that hold potential for valorization into bio-based chemicals, energy, and functional materials, thus reducing waste and enhancing process sustainability. Notably, optimized maleic acid pretreatment also achieved extensive lignin and hemicellulose removal, yielding high cellulose recovery and improved ethanol production through yeast fermentation, further demonstrating the importance of controlled lignin separation^6^. Taken together, these findings highlight that efficient lignin fractionation not only facilitates bioethanol production but also provides opportunities for lignin utilization, thereby improving the economic and environmental viability of integrated biorefinery systems^6^.

Resorcinol–formaldehyde (RF) resins are widely studied as polymeric precursors for the development of porous adsorbent materials^10^. When combined with lignin, a renewable and abundant aromatic biopolymer, RF-based composites exhibit enhanced structural stability, tailored porosity, and improved surface functionality. The incorporation of lignin not only reduces reliance on petrochemical feedstocks but also introduces additional oxygen-containing functional groups that promote stronger interactions with target pollutants^11^. During synthesis, the cross-linking of resorcinol and formaldehyde with lignin yields a three-dimensional polymeric network that can be carbonized or activated to produce highly porous adsorbents with large surface areas and tunable pore structures. These RF–lignin composites are particularly effective in adsorption applications, as they combine the robustness and tunability of RF resins with the sustainability and functionality of lignin, making them attractive candidates for wastewater treatment and environmental remediation.

With this motivation, a novel sulfonated lignin-resorcinol formaldehyde (LSR-F) composite has been synthesized using the lignin residue from bioethanol production to obtain a highly efficient adsorbent for the removal of toxic dye. The physicochemical properties and adsorption performance of the composite were investigated. Additionally, the kinetics, isotherms, and thermodynamics of the adsorption process were the reusability performance of the synthesized materials was evaluated, and the removal mechanism was explained.

## Materials and methods

### Materials

The lignin, a base adsorbent material, was a bio-industrial residue from the production of bioethanol using corn stover in a laboratory setup, which had previously been studied^12^. Crystal violet (C_25_H_30_ClN_3_) was supplied by Bio-Basic Canada Inc., assay 98%. Hydrochloric acid (HCl) (37%) was purchased from EMSURE for Analysis ACS, ISO, Reag. Ph Eur, Germany. Sodium chloride (NaCl) (99.9%) was bought from Universal Laboratories Pvt Ltd, India. Sodium hydroxide (NaOH) (98.5%) and sulfuric acid (H_2_SO_4_) (98%) were purchased from Pharaoh Imp. and Exp., Egypt. Formaldehyde (CH_2_O) (34–38%) was purchased from PioChem laboratory chemicals, Egypt. Sodium thiosulfate (Na_2_S_2_O_3_) was purchased from Orgachem Kimya San. Ve Tic., Turkey. Ethylenediaminetetraacetate (EDTA) was purchased from Park Scientific Limited, Northampton, United Kingdom. Span 80 (98%), resorcinol (99%), calcium carbonate (CaCO_3_), zinc chloride (ZnCl_2_), calcium chloride (CaCl_2_), and lithium chloride (LiCl) with purity ≥ 97% were provided by Sigma-Aldrich Co., St. Louis, MO, USA. Paraffin oil (99%) and acetone (C_3_H_6_O) (99.9%) were purchased from Alfa Chemical Group, Egypt. Hexanes (C_6_H1_4_) (≥ 95%) and ethanol (C_2_H_6_O) (99.8%) were purchased from Fisher Scientific, United Kingdom. Other chemicals were of the highest purity available commercially.

## Methods

### Fabrication of sulfonated lignin-resorcinol-formaldehyde (LSR-F)

**Fig. 1:**
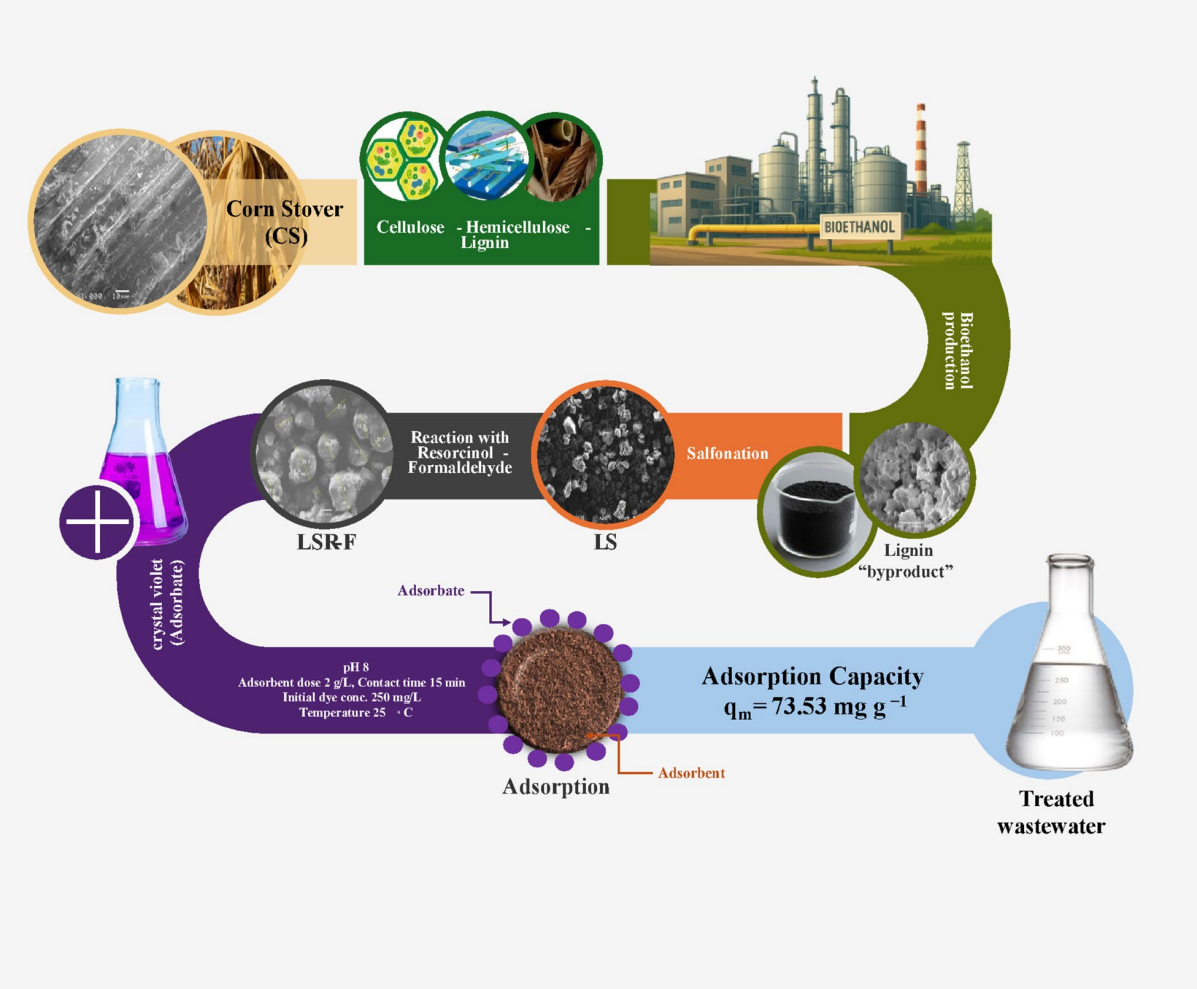
Schematic representation of the setup system for the synthesis of sulfonated lignin with resorcinol-formaldehyde (LSR-F) for the removal of toxic crystal violet dye in water.

The LSR-F hybrid composite was prepared from the lignin obtained from bioethanol production. Figure 1 shows the schematic representation of the fabrication of a hybrid composite for the removal of CV dye from an aqueous solution. The overall procedure for the synthesis of LSR-F is presented through three determinant steps as follows:

(i) ***Lignin from bioethanol production (L)***: Corn stover (CS) and 2% maleic acid are the feedstock for the pretreatment process in bioethanol production. Afterward, the treated corn stover underwent enzymatic hydrolysis to dissolve cellulose. Following the enzymatic process, the remaining suspension, considered a byproduct, contains a substantial percentage of lignin. Lignin was washed with distilled water to remove impurities and unwanted materials. It was kept in a furnace with hot air at 105 °C for 12 h for drying before being milled in a grinder (Kleinfeld Labortechnik, Cullati MFC grinder CZ 13, Gehrden-Germany) to a fine powder. The prepared materials were stored in sealed bottles at room temperature and labeled as (L).
(ii) ***Functionalized lignin by sulfonation (LS)***: The preparation of LS was illustrated in Fig. 1. A 10 ml solution of 0.1 M sodium thiosulfate was dissolved in 10 ml of acetone and heated at 55˚C under reflux and stirring (450 rpm) using paraffin oil. The mixture was left for 30 min. One gram of (L) was added to the solution at 55˚C for 30 min., stirring and refluxing to get the sample labeled as LS.
(iii) ***Sulfonated lignin-resorcinol formaldehyde (LSR-F)***: The LSR-F was synthesized by inverse emulsion sol-gel polymerization of resorcinol and formaldehyde in the presence of sulfonated lignin using Span 80 as a surfactant. Span 80 (S) was first dispersed in 80 ml of n-hexane and heated at 65 °C under reflux and stirring (450 rpm). Then, a mixture containing resorcinol (R), formaldehyde (F), and water (W), previously gelled at 65 °C for 1 h, was drop-wise into the solution of Span 80 and n-hexane, followed by adding the previously prepared solution of (LS). The obtained mixture was cured under the same conditions with vigorous stirring at 65 °C and covered for 24 h. After cooling to room temperature, the mixed solution was transferred into a sealed Teflon-lined stainless-steel autoclave and heated at 120 °C for 12 h, followed by natural cooling to room temperature. The solid product was washed with deionized water and acetone, respectively. Then, the obtained product was soaked in acetone for 5 days, changing the acetone every two days. Finally, the solids were dried in a vacuum drying incubator at 50 °C for 12 h.

### Characterizations of adsorbents

For the removal of lignin from corn stover in bioethanol production, the detailed yield of lignin, determination of cellulose, hemicellulose, lignin, and ash content of CS and L were described well in supplementary information S1. In addition, the proximate analysis, ultimate analysis, and higher heating value (HHV) of adsorbents are described well in supplementary information S2. The detailed physicochemical characterizations of the CS, L, LS, and LSR-F were investigated through TGA, CHNS elemental analyzer, FTIR, and SEM, as presented in supplementary information S3. To determine the surface charge of L, LS, and LSR-F, zeta potential and point of zero charge (pHpzc) are presented in supplementary information S4.

### Batch experiments for CV removal and adsorption mechanism

The detailed adsorption studies, kinetics, isotherms, and thermodynamics for designing the adsorption mechanisms and ionic strength are stated in the supplementary information (S5-S9).

### Reusability study

The reusability of the L, LS, and LSR-F adsorbents prepared for the adsorption of CV pollutants was evaluated by conducting adsorption-desorption experiments. Two desorbing solvent agents, water and 0.1 M HCl, were employed for comparison. Following the adsorption process, a mass of 0.05 g of L, LS, and LSR-F adsorbents loaded with CV were separated from the solution using filter paper (FP). The separated adsorbents were mixed with a 25 ml desorption solvent and agitated for 30 min at 200 rpm. After desorption, the material was separated again using FP and reloaded with CV dye through the adsorption process. This cycle of adsorption and desorption was repeated for a total of 10 cycles. Desorption capacity and efficiency were calculated using the following equations:

1 $${\rm q_{e} (desorp.) (mg/g) =V(C_{f}/m)}$$

2 $$={\rm (q_{e} (desorp.) /q_{e} (adsop.) ) *100}$$

Where m is the weight of spent adsorbent (g), V is the volume of solvent (L), and C_f_ is the concentration of CV dye desorbed by the regeneration solvent (mg/L).

Detection of formaldehyde during adsorption process.

The detection of formaldehyde during the adsorption process through sodium sulfite method (Hantzsch Reaction – indirect titration) as described in supplementary information (S10).

## Results and discussions

### Lignin by-product from bioethanol production

Even though corn stover (CS) is regarded as an economical and abundant lignocellulosic biomass for producing high-value products such as bioethanol, it requires pretreatment to break down its rigid structure and improve cellulose accessibility for enzymatic saccharification, consequently leading to a high bioethanol yield. Previous studies have shown that lignin is the major inhibitor of enzymatic saccharification in CS, unlike other herbaceous plants such as rice straw, where hemicellulose is the predominant inhibitor. An optimal pretreatment reduces lignocellulosic recalcitrance, making the biomass more accessible to enzymes and enabling higher hemicellulosic sugar recovery with fewer inhibitors^13^. Physical pretreatment mainly disrupts the structure, while chemical pretreatment removes or separates components, and their combination enhances saccharification efficiency. In the case of CS, pretreatment with maleic acid disrupts glycosidic linkages between hemicellulose and lignin, leading to hemicellulose dissolution and improved enzymatic accessibility of cellulose.

**Fig. 2:**
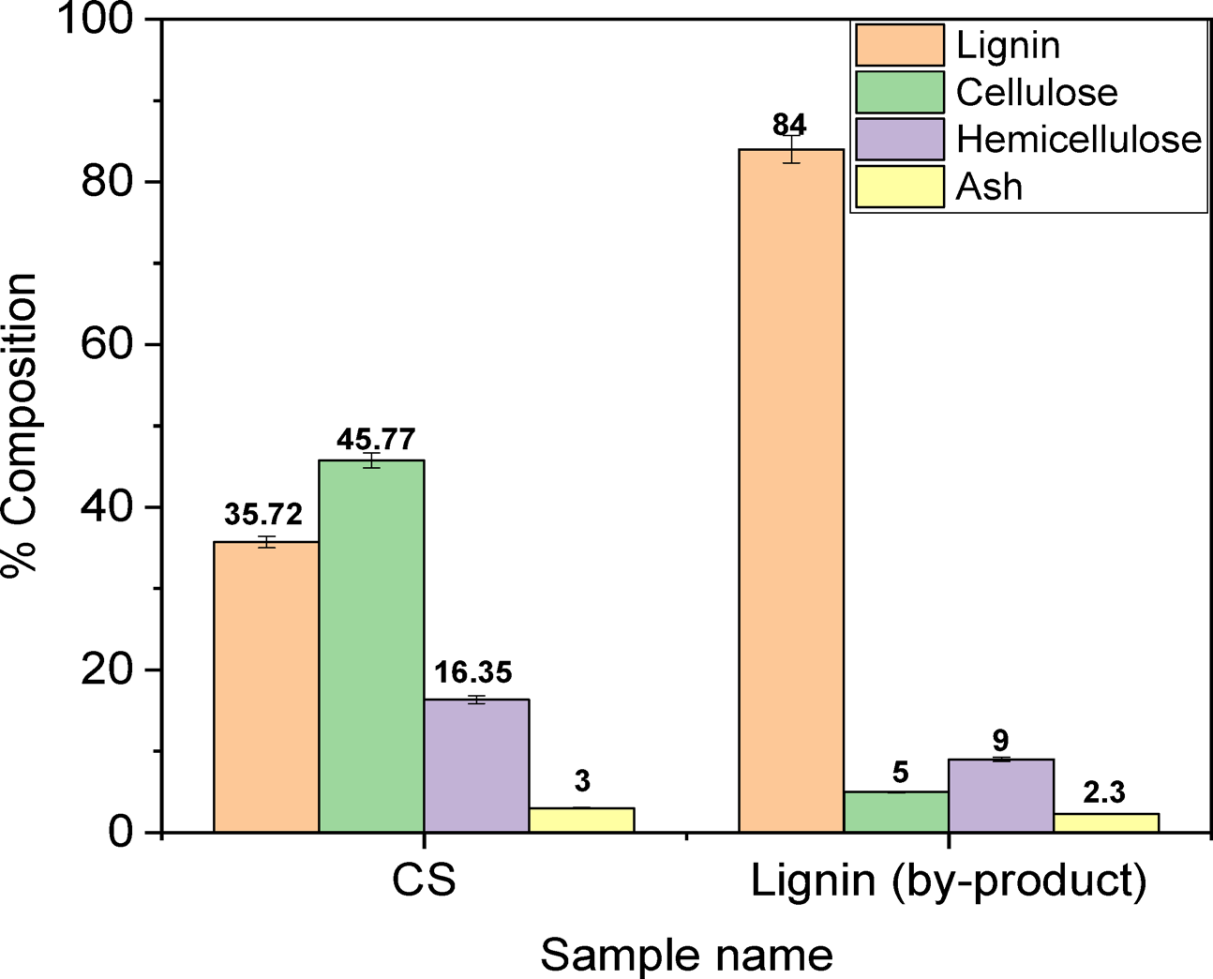
Chemical composition of CS and L.

The percentages of lignin, cellulose, and hemicellulose were determined for two primary materials in this study: CS and L. Figure 2 shows that the percentage of cellulose dropped from 45.77% in CS to 5% in L. In contrast, the percentage of lignin grew significantly from 35% in CS to 84% in L, while the amount of hemicellulose fluctuated around 10%. The decrease in cellulose and hemicellulose content is attributed to their composition of monomeric sugars with unstable glycosidic bond linkages, which tend to rupture under acidic conditions, resulting in breakdown and soluble sugars that serve as substrates for enzymatic action, leaving lignin as a byproduct during bioethanol production^6^.

### Physicochemical properties of LSR-F

The extracted lignin (L) is reacted with sodium thiosulfate to form sulfonated lignin (LS). Then, it is reacted with a resorcinol-formaldehyde derivative to form sulfonated lignin-resorcinol-formaldehyde (LSR-F) as an adsorbent.

Proximate analysis for moisture content (MC), volatile matter (VM), fixed carbon (FC), ash contents, and higher heating values (HHV) of samples L, LS, and LSR-F is presented in Table 1. Overall, the percentage contents of MC, VM, FC, and ash contents of sample LSR-F were within the literature range^14^. Moisture levels in all samples ranged from 1.4% to 2.9%, which is similar to results for other lignins^14^. The ash content decreased from 3.9% in the untreated lignin to 3.2% and 2.5 following the LS and LSR-F treatments, respectively. In large scale, the ash content can increase transportation and processing costs, potentially leading to slag deposits that hinder heat transfer and cause corrosion. In this study, LS showed a low level of carbon content, attributed to the success of the sulfur reaction. LSR-F had the lowest VM% and the highest FC%, making it better suited for adsorption applications due to its higher HHV and greater stability. The carbon content in LSR-F is higher than that in CS, which may be related to the generation of functional groups like hydroxyl groups that are beneficial for the adsorption process.

**Table 1:** Proximate analysis for CS, L, LS, and LSR-F adsorbents.

Sample	MC %	Ash %	VM %	FC %	HHV (MJ/Kg)
**CS**	10.09	2.2	70.63	17.08	13.11
**L**	1.5	3.9	30.87	63.73	14.1861
**LS**	2.9	3.2	35.495	58.405	8.0892
**LSR-F**	1.4	2.5	28.819	67.281	15.5348

Table 2 demonstrates the elemental analyses of the CS, L, LS, and LSR-F adsorbents using the CHNS elemental analyzer. LS and LSR-F have higher sulfur content, exceeding 3.68% and 1.45%, respectively, compared to L, which has 0.1%. This increase is due to the sulfonation process, consistent with previous research^15^. The carbon content in CS was higher because it contained lignin, cellulose, and hemicellulose. Conversely, in L and LS, the carbon content decreased as a result of the acidic treatment applied to CS and the conversion of cellulose and hemicellulose into soluble sugars during bioethanol production^6^. In contrast, the reaction of LSR-F with resorcinol and formaldehyde increased its carbon content^11^. The L sample revealed the lowest H/C atomic ratio, indicating the highest aromaticity and most developed aromatic structure. In contrast, the higher H/C ratios in LS and LSR-F suggest a greater presence of initial organic components, such as lignin and polymeric CH_2_ within the aromatic core. The changes in H/C and O/C observed in the CS sample suggested that the structural characteristics were affected by the physicochemical pretreatments and proved the high efficiency of the delignification process^6^.

**Table 2:** Elemental composition of the CS, L, LS, and LSR-F by CHNS.

Sample	C %	H %	*N* %	S %	O %	H/C	O/C
**CS**	60.24	5.64	0.04	0.02	34.06	0.094	0.565
**L**	45.41	3.37	0.53	0.10	50.59	0.074	1.114
**LS**	29.92	3.411	0.57	3.68	62.42	0.114	2.086
**LSR-F**	44.14	4.618	0.66	1.45	49.13	0.105	1.113

**Fig. 3:**
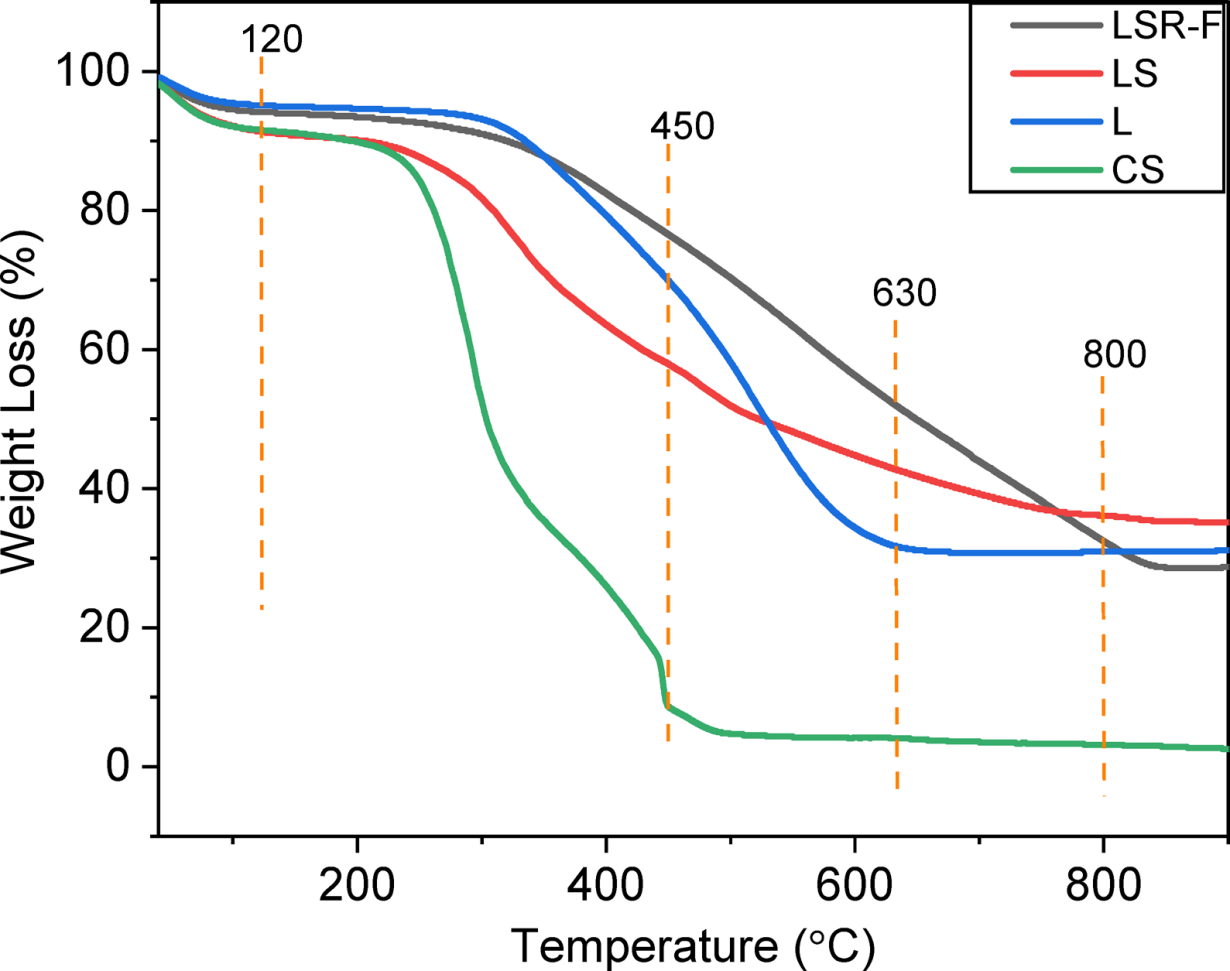
TGA of CS, L, LS, and LSR-F.

To evaluate the effect of the chemical pretreatment of CS as well as the effect of sulfonates and resorcinol-formaldehyde reaction for L, we conducted decomposition analyses of the adsorbents using TGA. In general, it is evident that the adsorbent’s decomposition is represented by four stages of weight loss, as illustrated in Fig. 3. Precisely, it was discovered that every examined sample had a distinct proportion of water and lignocellulosic fiber humidity that evaporated in the first stage within the temperature range of 25 to 120 °C, representing 8.35, 4.85, 8.61, and 5.84% in CS, L, LS, and LSR-F, respectively, corresponding to the drying stage with non-dissociative water molecules that are physically adsorbed, along with surface water molecules forming hydrogen bonds^16^. The second stage, within the temperature range of 120 to 450 °C, representing the weight reduction for the CS, was 83.04%, corresponding to the breakdown of the crystalline structure and the degradation of the biomass, resulting in an enhanced amorphous structure and reduced polymerization level^17^. This significant weight loss can be primarily attributed to the cleavage of glycosidic linkages present in cellulose, hemicellulose, and certain components of lignin^18^, confirming the release of volatile hydrocarbons and flammable gases, including C_x_H_y_, CO, and CO_2_. These gases arise from the evaporation of diverse oxygen functional groups present in their structure, exhibiting varying thermal stabilities^19^. In the same range, from 120 to 450 °C, the rapid thermal decomposition amounted to 25.26%, 33.44%, and 17.73% in L, LS, and LSR-F, respectively. The decomposition percentage increased in LS as a result of the sulfonation reaction, while the percentage decreased again in LSR-F due to the reaction with resorcinol and formaldehyde, which formed hydrocarbons. The third stage, within the temperature range of 450 to 630 °C, representing 4.53, 38.06, 15.01, and 24.23% in CS, L, LS, and LSR-F, respectively, corresponds to the breakdown of the cross-linked phenol polymer (hydroxyl and methoxy), α-O-4, β-O-4 linkages, C–O–C, and C = O bonds in CS, L, LS, and LSR-F, causing the emission of CO^21,22^. Finally, the fourth stage, within the temperature range of 630 to 800 °C, represents 0.91%, 0.87%, 6.84%, and 19.69% in CS, L, LS, and LSR-F, respectively, corresponding to the complete breakdown of crystalline and carbonaceous matter^22^. Within this stage, the degraded volatile compounds are derived from phenolics, ethers, and aldehydes. Notably, the LS and LSR-F samples are highly thermally stable due to the presence of the sulfonate functional group and the formation of stable hydrocarbons from resorcinol-formaldehyde reactions^13^. Overall, all composites are highly thermally stable because of physicochemical modification in comparison with CS.

The proposed interaction assumption is formulated using FTIR spectroscopy. The absorption peaks in the CS sample showed that it had a complex and heterogeneous structure in which cellulose fibers are clumped with lignin and hemicellulose, and their functional groups appeared effectively. For example, a broad band at 3000–3500 cm^− 1^ can be attributed to -OH stretching of cellulose, hemicellulose, and lignin^23^. The peak at 2920 cm^− 1^ is attributed to the presence of C–H groups of the side chains in CS^25^. Upon the addition of 2% maleic acid into CS for bioethanol synthesis, a lignin by-product was generated, leading to the splitting of the peak into two distinct peaks at 2920 and 2850 cm^− 1^. These peaks are attributed to the presence of the C–H group within the side chains of lignin and the stretching vibrations of the carboxylic acid group introduced by the maleic acid addition^6^. Additionally, on both LS and LSR-F adsorbents, the spectral peaks remained split within the same spectral region, suggesting the presence of carboxylic acid groups originating from the reaction involving Span 80 (derived from oleic acid)^25^. The peak at 2350 cm⁻¹ observed in all samples is attributed to the –OH stretching vibration of hydroxyl groups. Notably, this band is more intense in the LS sample, suggesting a greater introduction of oxygen-functional groups^9^. The peak at 1640 cm^− 1^ attributed to C = C aromatic skeletal stretching vibrations and C = O stretching of lignin was located in all samples^9^. The peak at 1437 cm^− 1^ is attributed to the methylene = CH_2_ stretching in CS; however, the stretching in L, LS, and LSR-F was weakened and shifted, suggesting the destruction of the intramolecular hydrogen bond^9^. Moreover, peaks around 1077 cm^− 1^ are attributed to the stretching of the ether group C–O–C in (dibenzodioxin) α-O-4 and (Aryl glycerol ether) β-O-4 linkages to lignin^26^. The band at 790 cm^− 1^ is attributed to the out-of-plane bending of the C–H bond in L, LS, and LSR-F, attributing to the substitution pattern on a benzene ring^27^. The stretching vibration band at 617 cm^− 1^ is attributed to sulfate (SO_4_^2−^) in LS and LSR-F, indicative of the reaction with sodium thiosulfate^27^. These spectral features collectively confirm the successful incorporation of sulfonated functional groups and the reaction with resorcinol-formaldehyde, which are directly responsible for the enhanced adsorption of cationic dyes. Moreover, this interpretation aligns with recent life cycle assessment (LCA) studies on lignin-derived adsorbents, which demonstrate that the functionalization of waste lignin into sulfonated lignin-RF adsorbents can be achieved under mild conditions with reduced energy input, thereby supporting both the economic and ecological feasibility of the present approach^28^.

**Fig. 4:**
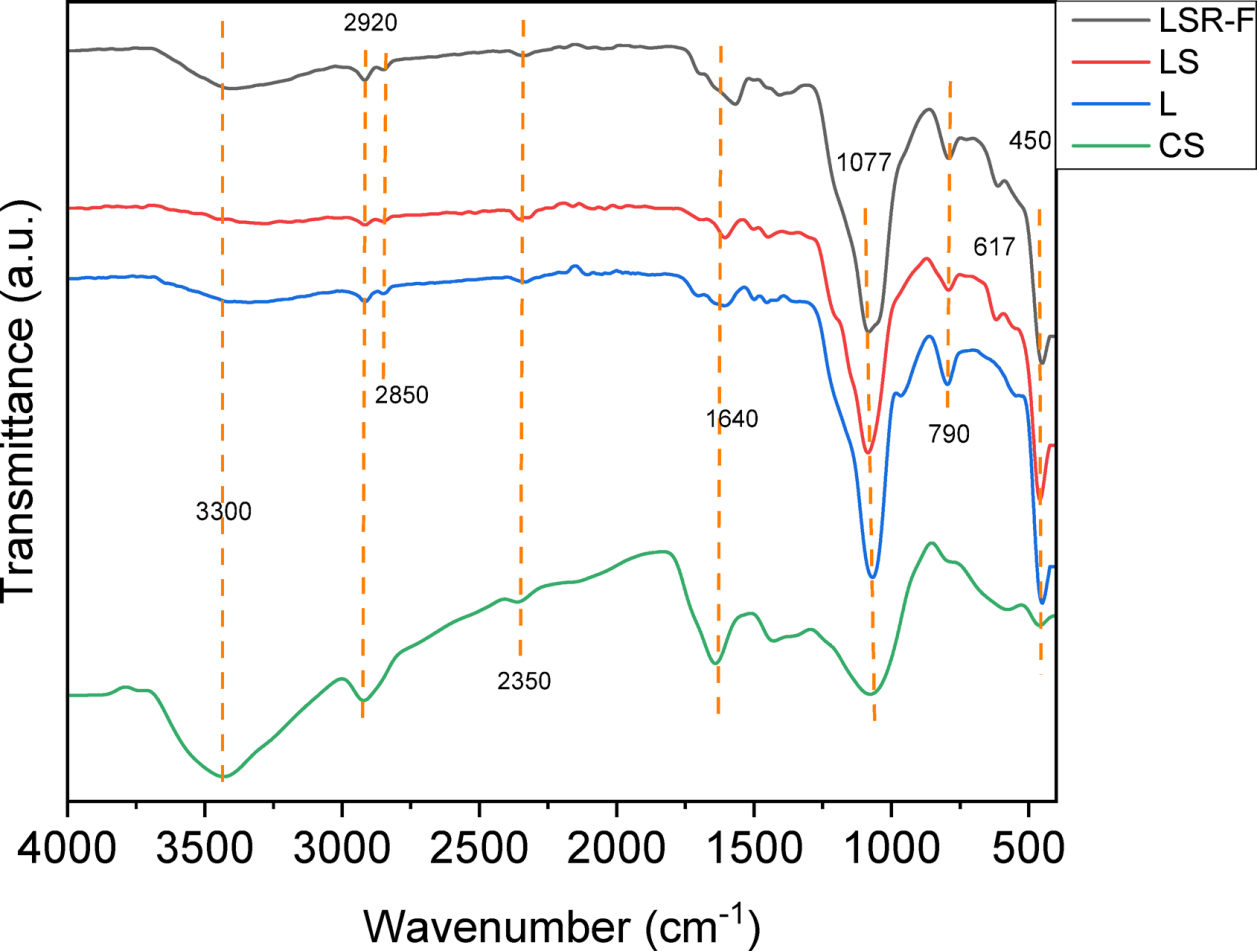
FTIR of CS, L, LS, and LSR-F.

As seen in Fig. 4, the SEM images revealed notable variations in surface morphology between the CS, L, LS, and LSR-F. In Fig. 4a, CS had a smooth, compact structure with thick-walled fiber cells, lacking pores or cracks. This suggests significant lignin coverage and cellulose protection, contributing to biomass recalcitrance^29^. L, LS, and LSR-F exhibited clear differences in their surface compared to CS. In Fig. 4b, the lignin particles also exhibited a smooth texture with a collection of irregular aggregates of varying particle sizes and pores, attributed to moisture loss during heat drying^30^. When compared to untreated L, SEM imaging of the sulfonated lignin surface (LS) showed different shades of gray, indicating different textures and densities with intercellular gaps (Fig. 4c). The lignin cracked into several separated irregular shapes as a result of the interaction of sodium thiosulfate groups, which changed the chemical composition and encouraged intermolecular interactions^31^. In Fig. 4d, a semi-spherical structure (with a mean diameter of 20 micrometers) of LSR-F is shown. A cumulative structure with a rough texture was distinguished by differences in surface height after sulfonation and the resorcinol-formaldehyde reaction^32^. The sulfonation and resorcinol-formaldehyde reaction may have potentially impacted lignin’s performance in adsorption by increasing reactivity and surface area, respectively^33^.

### Formation mechanism of LSR-F

**Fig. 5:**
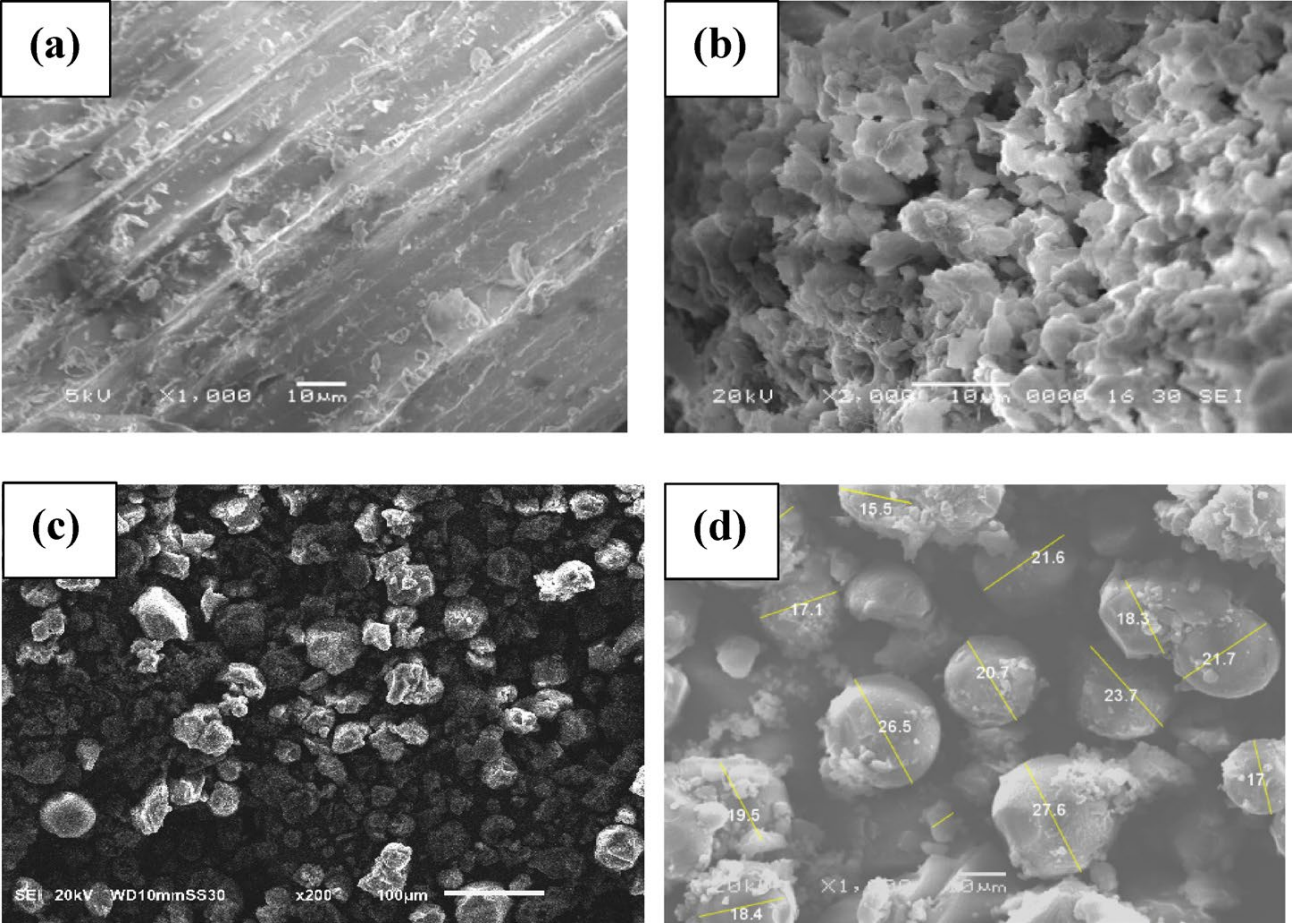
SEM of **(a)** CS **(b)** L** (c)** LS, and **(d)** LSR-F.

Figure 5 indicates the formation mechanism of LS and LSR-F adsorbents. At step **(1)**, sulfonation occurs through the nucleophilic attack of the sulfur atom from sodium thiosulfate to the carbon atom of the carbonyl group in acetone. This leads to the formation of a tetrahedral intermediate, which eventually rearranges to form sulfur trioxide and a side product. The substitution of a sulfur group on lignin’s aliphatic hydroxy groups occurs through the addition reaction. The reaction primarily occurs on the carbon of the α position to form sulfonated lignin (LS)^34^. At step **(2)**, the resorcinol-formaldehyde derivative (Resorcinol hydroxymethyl derivative) is prepared from the reaction of formaldehyde with resorcinol in the presence of water at 65^◦^C in a dark bottle to avoid light degradation^10^. During step **(2)**, the following stages occur: **(a)** electrophilic substitution reaction to form the resorcinol-formaldehyde derivative. The high reactivity of resorcinol in this stage increases due to the electron density of the aromatic ring at positions 2, 4, and 6, but the reaction mainly occurs at positions 4 and 6 due to steric hindrance at position 2; **(b)** condensation reaction for the resorcinol-formaldehyde derivative to form methylene (-CH_2_-) bridges; **(c)** ether (-CH_2_-O-CH_2_-) bridges; and **(d)** formation of the cross-linked structure of organic gel (Product I). At step **(3)**, the resorcinol-formaldehyde derivative compound (Product I) reacts with n-hexane (non-polar solvent) and Span 80 (reaction stabilizer) at 65^◦^C for 1 h through the condensation system to form (Product II). Then, sulfonated lignin will react with (Product II) at 65◦C for 24 h to make the encapsulation of sulfonated lignin xerogels, producing LSR-F adsorbent solution (Product III). Finally, the mixture is sealed in a Teflon-lined stainless-steel autoclave under 120◦C for 12 h to precipitate the LSR-F adsorbent. The precipitate is separated from the rich carbon chains solvent by centrifugation at 5000 rpm for 5 min. The separate particles of LSR-F undergo a purification process by washing with deionized water and acetone.

## Adsorption study

### Reactivity of LSR-F

**Fig. 6:**
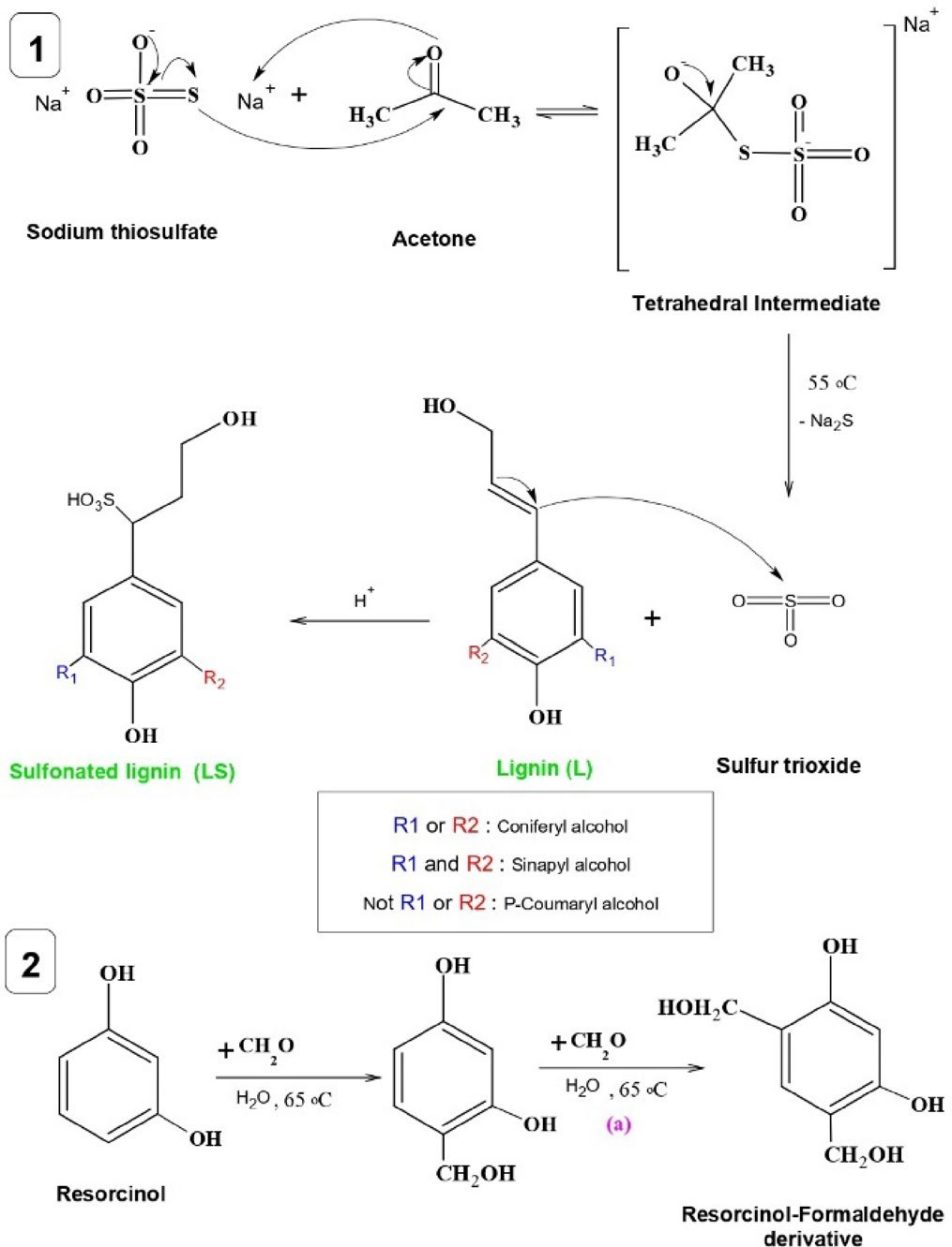

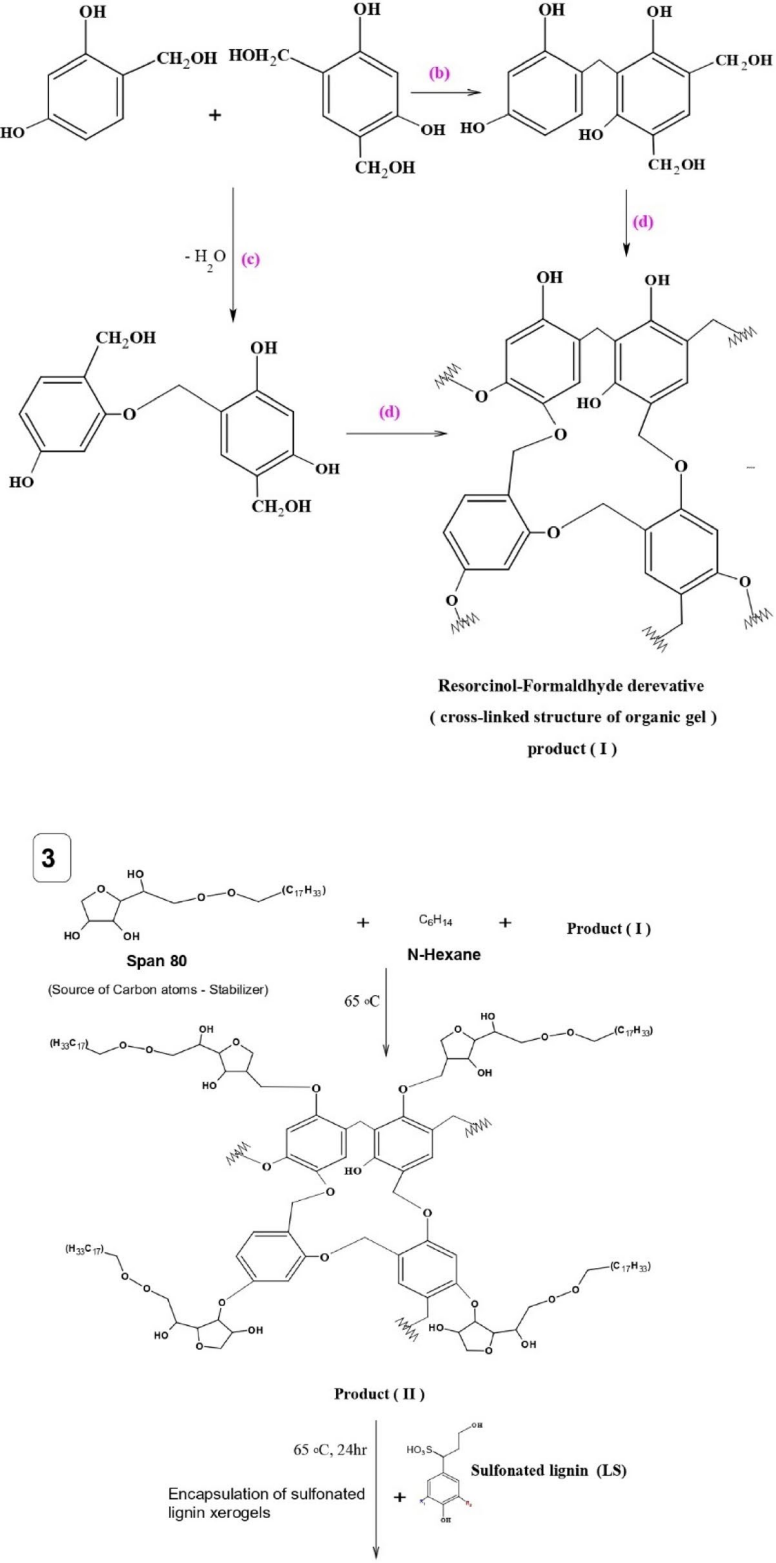

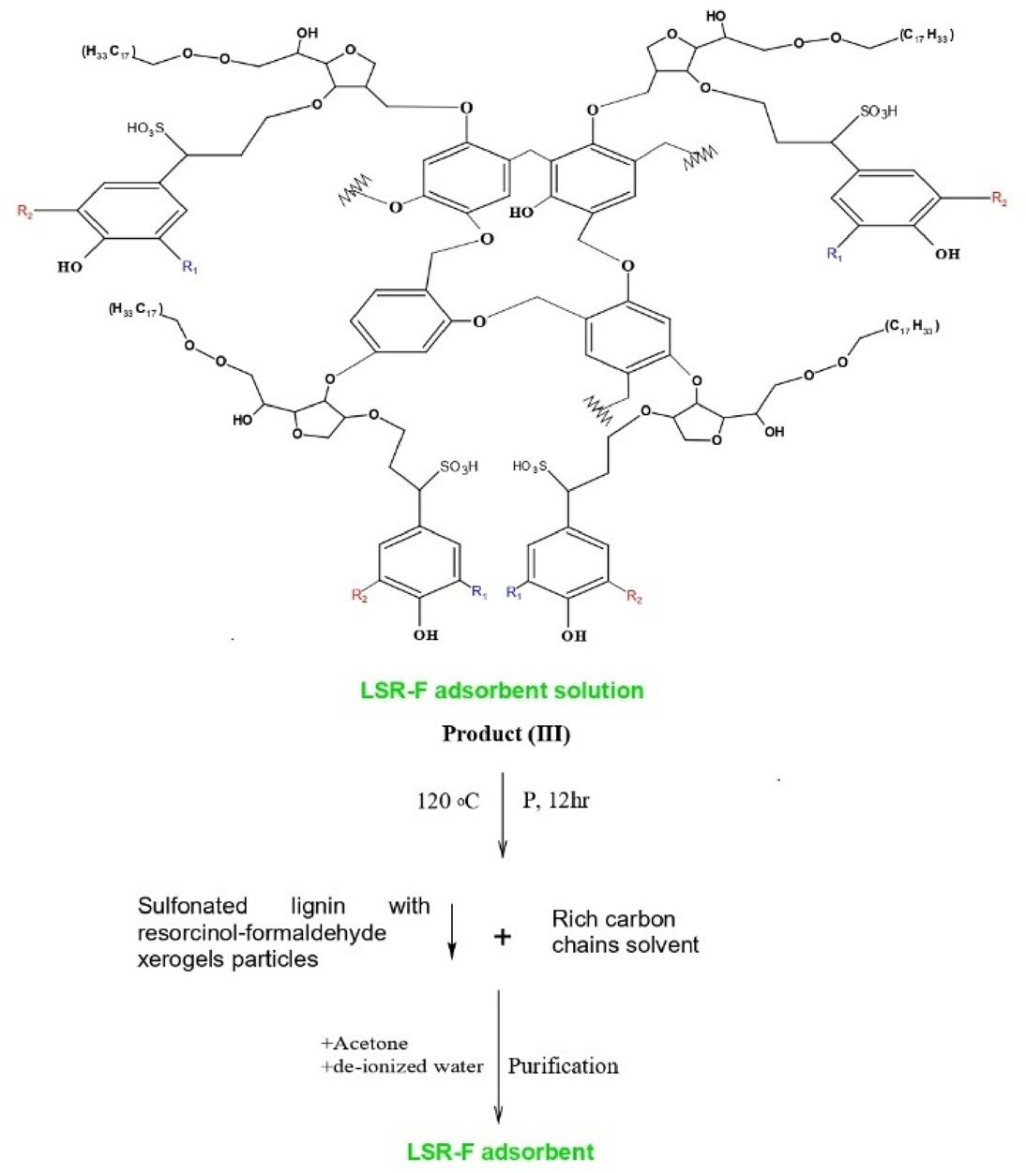
Formation mechanism of LS and LSR-F adsorbents

From Fig. 6, the results indicate that the order of CV removal efficiency was LSR-F > LS > L. As a result, LSR-F was the best sample for removing CV, while L exhibited the lowest removal efficiency. It is clear that the CV dye removal improved as a result of sulfonation and/or reaction with resorcinol-formaldehyde.

Investigation of the effect of various parameters on the adsorption of CV dye onto the surface of LSR-F samples was affected by various factors in order to estimate the optimum conditions and design the adsorption mechanism through kinetics and isotherms as follows:

### Contact time and kinetics

The contact time between the CV dye and adsorbents has a strong influence on the adsorption process in different time intervals ranging from 3 to 120 min. As illustrated in Fig. 6, the adsorption of CV dye onto L, LS, and LSR-F underwent two steps. The first stage occurred at the highest percentage of dye removal for 15 min, where the adsorption efficiency reached 62%, 72%, and 85%, respectively. Earlier researchers used extracted lignin named PTA obtained from palm tree trunk to adsorb crystal violet dye and found that 1 g of adsorbent removes a percentage below 60% at 30 min^35^. The fast kinetics of the adsorption process were indicated by the rapid equilibrium observed, where the CV quickly occupied the available adsorption active sites on the L, LS, and LSR-F until they reached equilibrium. This quick decolorization can be attributed to three factors: the abundance of unoccupied active sites on the adsorbent surface^37^, the driving force indicated by the concentration difference between the CV in the solution and the surface of the adsorbent. The second stage was the slowest in adsorption, which required around 60 min for LSR-F to reach 85.8% removal.

Beyond the 15-minute mark, there was a simultaneous decrease in the adsorbents, as well as the increase in the number of free adsorption sites and CV concentration. The gradual slowing down of the adsorption process could be attributed to the formation of a monolayer on the adsorbent surface, as vacant sites became less available after reaching equilibrium. This could be explained by the fact that large pore spaces were occupied to induce rapid removal, while smaller pore spaces gradually filled to cause delayed removal^37^. After analyzing the data, it was observed that although the adsorption increased over time, the percentage removal did not exhibit significant improvement. Therefore, it can be concluded that the equilibrium.

is 15 min.

**Fig. 7:**
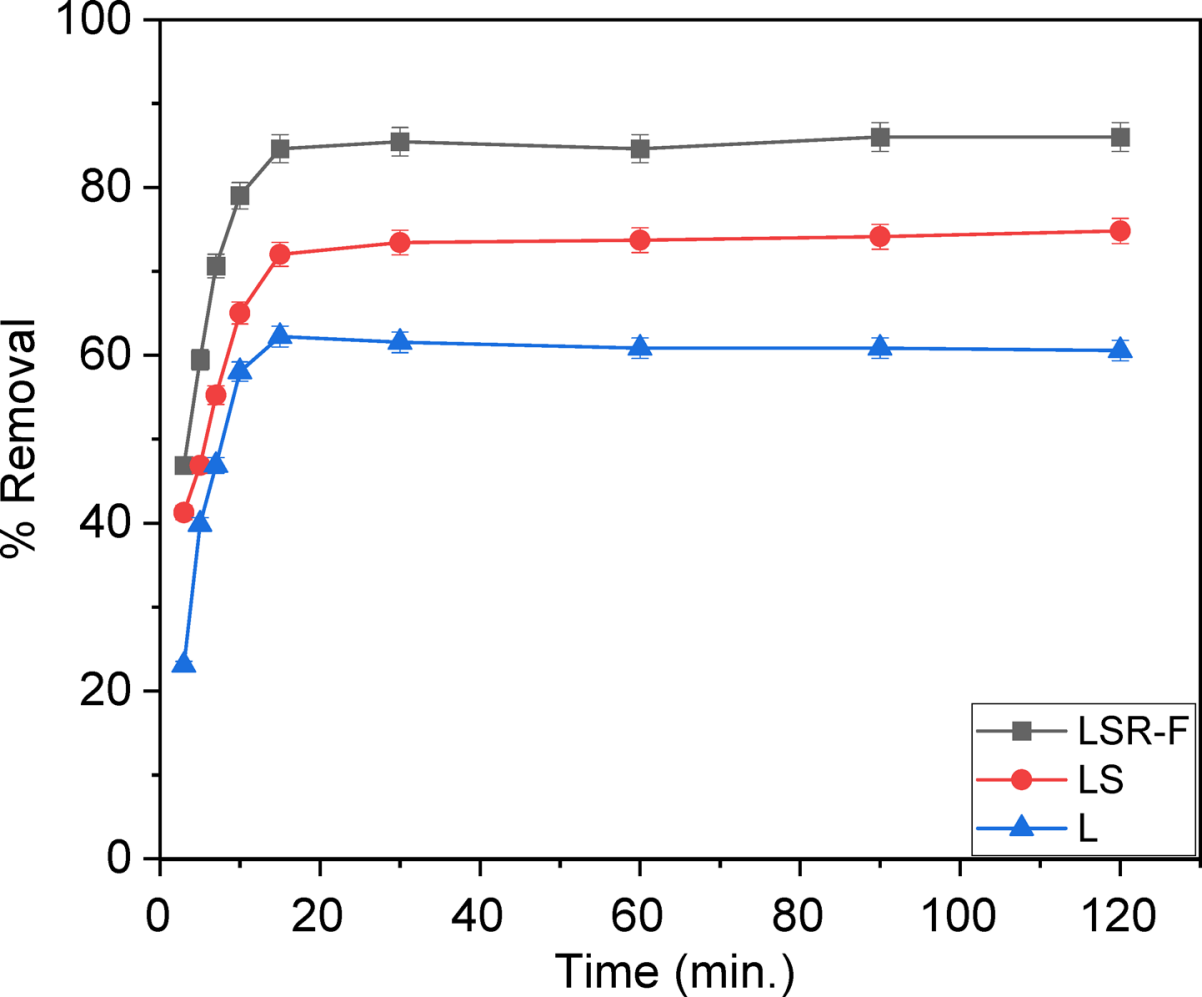
Effect of contact time on removal percentage for adsorption of CV dye onto L, LS, and LSR-F adsorbent [contact time = 3–120 min, concentration of dye = 250 mg/L, dose = 0.05 g, pH = 7, T = 25 ◦C].

Adsorption kinetics analysis describes the rate at which solutes are absorbed at the solid-solution interface, providing insights into reaction mechanisms. This study examined CV dye adsorption on LSR-F adsorbent using five kinetic models: pseudo-first-order, pseudo-second-order, intra-particle diffusion, Boyd, and Elovich. The kinetics of CV dye onto LSR-F was described using the linear forms of these models (Table S1). The agreement between the experimental data and the model’s calculated values is represented by the R² value as the correlation coefficient, as shown in Fig. 7.

The rate of adsorptive interaction was examined using the Lagergren equation, a fundamental model that links the adsorption rate to the availability of unoccupied sites for solute binding. The calculated values of k_1_, q_e_, and R² for fitting the first-order rate model at different contact times are detailed in Table 3. The linear relationship for LSR-F adsorbent concentrations is illustrated in Fig. 7a, with an R² value is about 0.90. Thus, this model is not suitable to describe the entire sorption sites onto heterogeneous surfaces. However, the pseudo-second-order model recorded the highest satisfactory linear fitting, where the value of R² is higher than 0.99 (Fig. 7b). When examining the data, it was found that the adsorption of CV dye onto LSR-F adsorbent is controlled by the rate at which the adsorption process occurs. By comparing different kinetic models, the second-order model depicted in Fig. 7c presents a superior fit compared to the first-order equation, indicated by its higher R² value. These results confirmed that the sorption processes obeyed pseudo-second-order kinetics, as shown in Table 3, indicating a stronger agreement between the model and the actual data. This model implies that the adsorption process of CV dye onto LSR-F is based on the chemisorption process.

The movement of dye ions from the solid interface to the interior of solid particles is vital for the rate of metal ion adsorption. While the pseudo-second-order equation best fits the experimental data, it does not accurately predict the diffusion mechanism. Evaluating the initial adsorption rate helps assess intra-particle diffusion, which involves the movement of adsorbate from the bulk solution to active sites within the solid, controlled by external mass transfer, pore diffusion, or both. The slowest step in this process is either pore or film diffusion, which determines the overall adsorption rate. This equation describes the intra-particle diffusion process illustrated in the supplementary materials. Figure 7c; Table 3 present the fitting of the intra-particle diffusion model to the adsorption of various concentrations of CV dye. It was noted that the plot of q against time is not consistently linear throughout the entire process; instead, it can be divided into two distinct zones. The CV adsorption on LSR-F adsorbent exhibits multiple stages. If the plot passes through the origin, intra-particle diffusion is the primary rate-limiting step; if not, multiple kinetic stages are involved. The analysis indicates a three-stage process. In the initial rapid phase, the plot shows a linear trend, indicating fast mass transfer as CV diffuses from the bulk solution to the adsorbent surface. The second linear trend reflects CV moving into the pores through intra-particle diffusion, with the rate increasing with initial CV concentration, marking a gradual adsorption stage. The third stage involves diffusion through smaller pores, leading to the establishment of the final equilibrium where intra-particle diffusion slows as all active sites are occupied.

In Fig. 7c, the non-zero intercepts suggest that while intra-particle diffusion contributes to CV adsorption on LSR-F adsorbent, it is not the only determining factor. Two mechanisms influence the intra-particle diffusion rate: pore diffusion within the pore volume and surface diffusion along the pore surfaces, both occurring simultaneously. Thus, the adsorption process may not be solely diffusion-controlled due to boundary layer resistance.

The movement of solute molecules from the bulk liquid to the solid surface is key in determining the rate-limiting step. To identify this slow step in the sorption process, kinetic data were analyzed using the Boyd equation (Table S1). The plot of against time is shown in Fig. 7d. The linearity of these plots helps determine if adsorption kinetics are controlled by liquid film diffusion or intra-particle diffusion, affecting metal sorption rates. While the plots yield high correlation coefficients R² (0.95), the non-zero intercepts confirm that CV adsorption on LSR-F follows the intra-particle diffusion model.

Elovich’s equation (Table S1) is often used to describe gas adsorption on solids and to explain heavy metal adsorption from aqueous solutions. This model characterizes the kinetics of the chemisorption process. The Elovich equation captures second-order kinetics, assuming an energetically heterogeneous solid surface. Parameters α and β are derived from the slope and intercept of the linear plot of versus ln(t), as shown in Fig. 7e; Table 3. The constant α indicates that higher initial adsorption rates with temperature may reduce the available adsorption surface. The results suggest that the diffusion rate-limiting significantly influences CV adsorption on LSR-F. Increasing adsorption temperature enhances the rate, consistent with activated chemisorption systems where high α indicates more available sites. Kinetic findings indicate that both film and pore diffusion affect adsorption mechanisms^12^.

The observation that the adsorption data of LSR-F fit well to the pseudo-second-order kinetic model suggests that the adsorption process may involve a combination of physical and chemical interactions. This dual compatibility indicates the possibility of a multi-step mechanism, where initial rapid physisorption on the external surface is followed by slower chemisorption or diffusion into internal pores. The pseudo-second-order model is associated with chemisorption. However, it is important to emphasize that kinetic models primarily describe the rate behavior and do not conclusively define the mechanism; thus, additional analyses such as isotherm modeling or spectroscopic characterization are recommended to confirm the nature of adsorption.

**Table 3:** Kinetic parameters and constants of CV dye onto LSR-F.

Model	Parameter	Unit	
Pseudo-first-order	q_exp_	(mg/g)	54.88
	K_1_	(1/min)	0.085
	q_theo_	(mg/g)	51.53
Pseudo-second-order	K_2_	(g/mg.min)	0.012
	q_theo_	(mg/g)	52.97
Intra-particle diffusion	K_dif 1_	(mg/g.min^0.5^)	10.14
	C1		16.14
	K_dif 2_	(mg/g.min^0.5^)	2.46
	C2		38.11
	K_dif 3_	(mg/g.min^0.5^)	0.79
	C3		45.30
Elovich	α	(mg/g.min)	0.04
	β	(g/mg)	0.21

### Dosage, initial concentration of CV dye, and isotherms

**Fig. 8:**
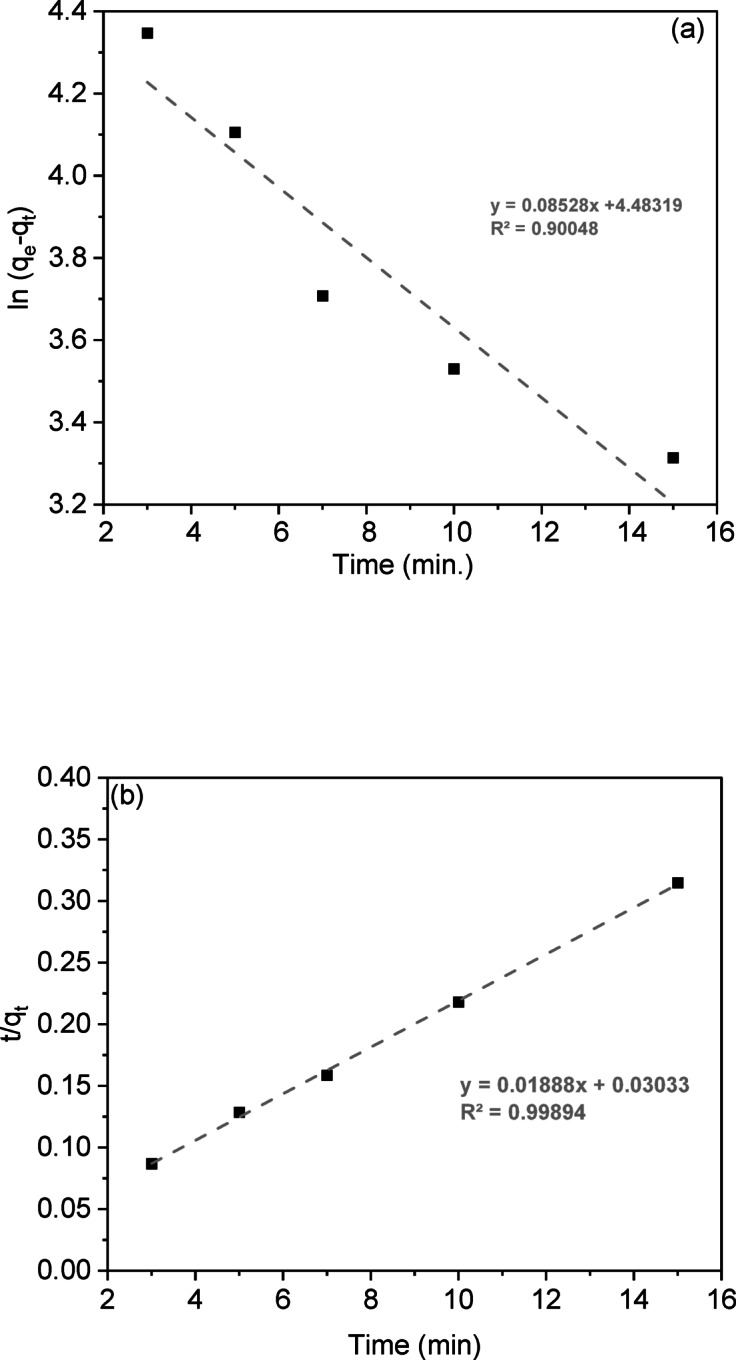

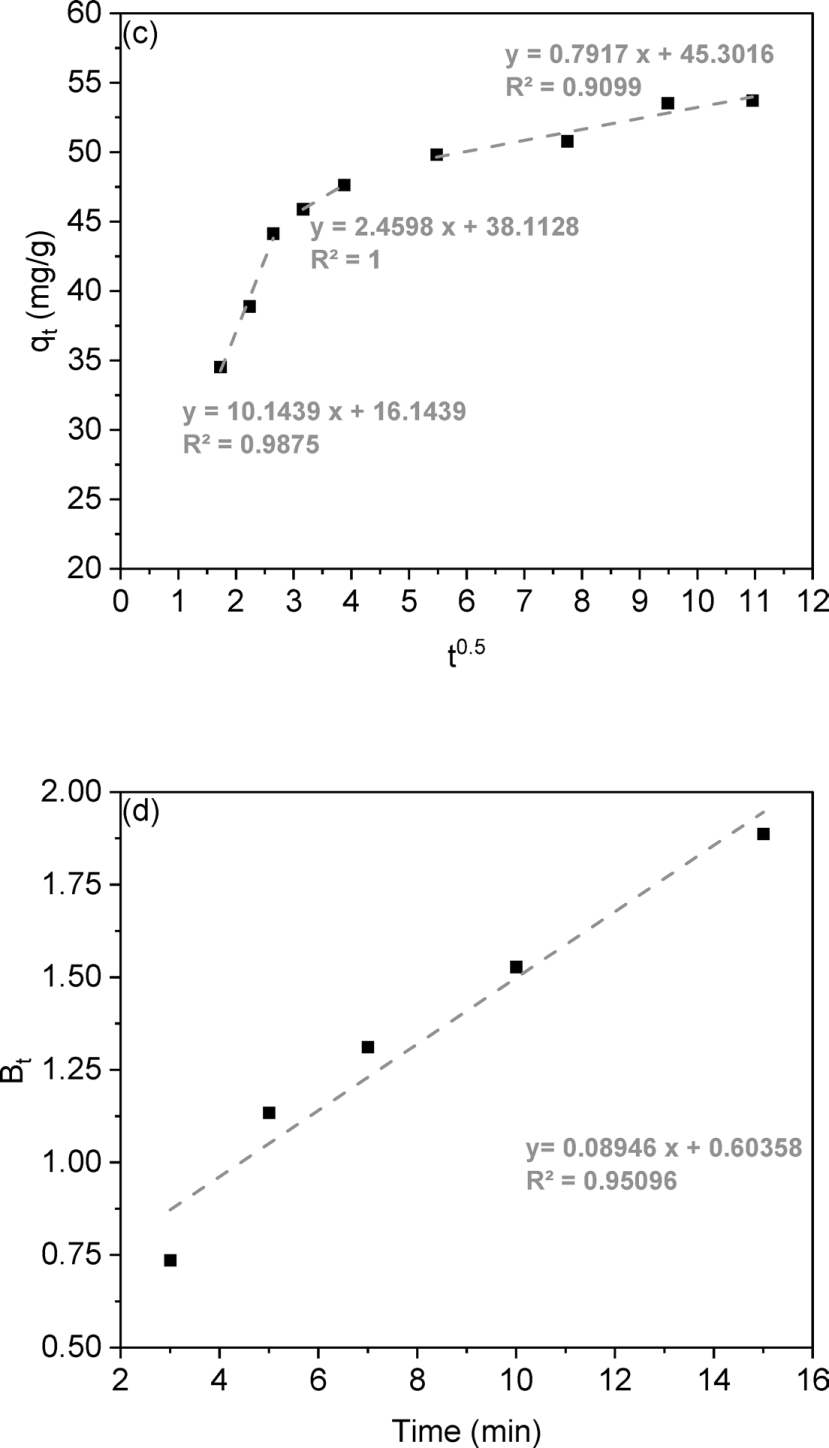

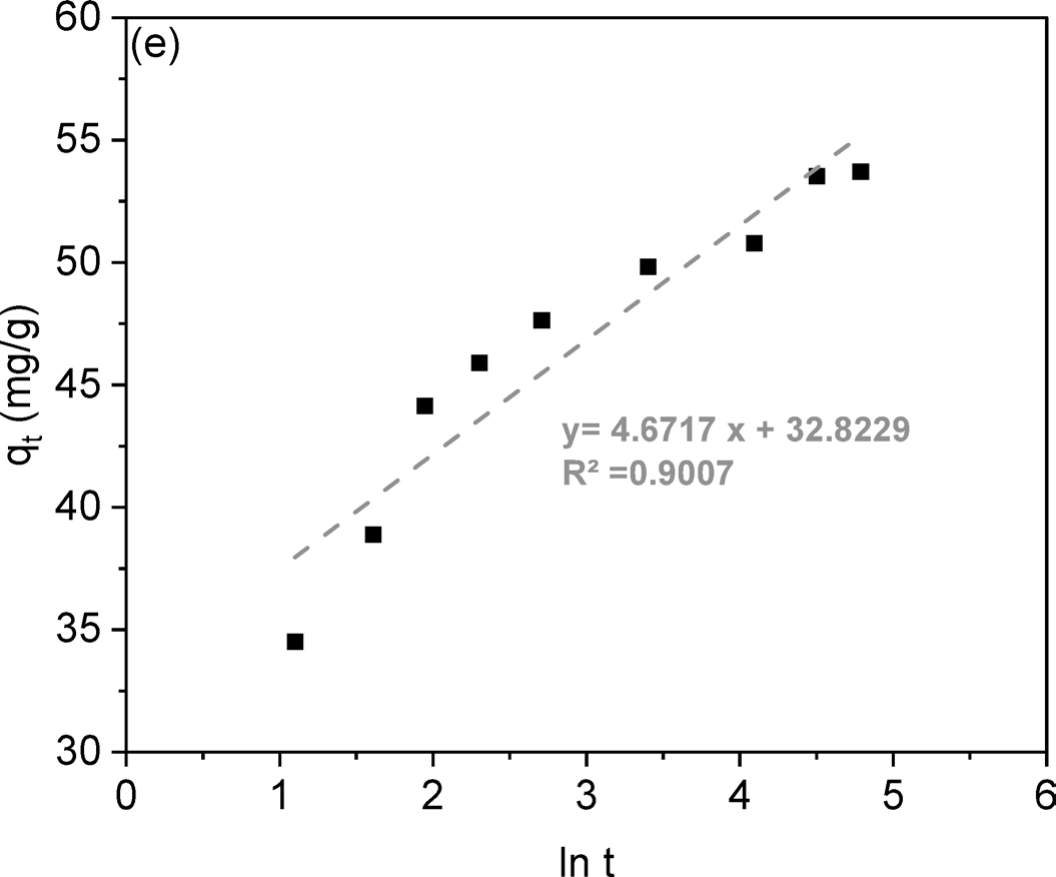
Kinetic plots for the adsorption of CV by LSR-F of **(a)** pseudo-first-order **(b)** pseudo-second-order **(c)**intra-particle diffusion, **(d)**Boyd, and** (e)** Elovich models.

The effect of adsorbent dose on the percentage removal of CV was investigated by introducing various masses of adsorbents (0.025, 0.05, 0.1, 0.15, 0.2, and 0.25 g) into a 25 mL CV aqueous solution containing 250 ppm of dye. The experimental conditions included a contact time of 15 min at room temperature, with an agitation speed of 200 rpm. The results, depicted in Fig. 8a, demonstrated a gradual increase in the percentage of CV removal, rising from 76% to 88% as the adsorbent dosage increased from 0.025 g to 0.25 g. This observed increase in dye removal may be attributed to the enhanced surface area, active sites, and pores of the number of unsaturated sites^38^. However, once the percentage removal reaches a relatively constant level, further increases in adsorbent weight may lead to the accumulation of particles, thereby blocking the active sites. This hindrance impedes the access of CV molecules to the active sites, resulting in a plateau in removal efficiency. In the present study, the optimal dosage was determined to be 0.1 g. Similar trends were also observed by other researchers in their investigations on CV removal from aqueous solutions using coffee husk as a cost-effective adsorbent^39^.

By studying the effect of varying the initial concentration on the dye removal percentage and consequently on the degree of water discoloration due to the pollutant, the initial concentration was varied between 25 and 500 mg/L. It was found that the adsorbents were capable of removing more than 90% of the dye at concentrations up to 150 ppm. However, with increasing concentrations, the removal efficiency decreased, reaching 88% at a concentration of 250 mg/L, as illustrated in Fig. 8b. High concentrations provide a strong driving force to overcome all resistances to the mass transfer of CV dye between the aqueous and solid phases. In contrast, at lower concentrations, all dye molecules present in the adsorption medium can interact with the surface, making the ratio of available binding sites crucial for selecting the initial concentration^40^.

Analyzing isotherm data is essential for evaluating an adsorbent’s adsorption capacity. Adsorption isotherms are based on two key principles: site homogeneity, where each site is equivalent, and site independence, where a site’s binding capability remains unchanged regardless of neighboring site occupancy^4^. The adsorption process between liquid and solid phases at equilibrium is crucial for understanding the adsorbate transfer mechanism and optimizing adsorbent use. The linear forms of Langmuir, Freundlich, and Temkin isotherm models, along with their plots, are given in Table S2, and the linear plotting of these models was investigated in Figs. 8d-f. The isotherm parameters calculated from the slopes and intercepts are presented in Table 4.

The Langmuir model explains adsorption through a monolayer mechanism on uniform sites with consistent energy. It states that once a site is occupied, further adsorption is prevented. Higher values of the Langmuir constants indicate more favorable adsorbents. The model equation is represented in Table S2. From Fig. 8c, the correlation coefficient for the removal of CV dye adsorption onto LSR-F at initial concentrations exceeds 0.99, indicating a strong model fit. Table 4 shows that the calculated monolayer adsorption capacities (q_m_) for CV dye adsorption onto LSR-F was 73.53 mg/g. This indicates a strong electrostatic attraction between the CV dye and the prepared LSR-F adsorbent binding sites. The suitability of the Langmuir isotherm indicates homogeneous, monolayer adsorption on the adsorbent surface, characterized by a finite number of identical sites, no transmigration of adsorbate across the surface, and uniform adsorption energies, confirming that electrostatic interaction predominates in this process. To assess the feasibility of the Langmuir model, the separation factor (R_L_) is estimated. These values serve as indicators of the type of isotherm behavior observed: irreversible (R_L_ = 0), favorable (R_L_ < 1), linear (R_L_ = 1), or unfavorable (R_L_ > 1). The values of R_L_ in Table 4; Fig. 8d are between 0 and 1, confirming the favorability of the Langmuir model for CV ion adsorption at different initial concentrations of CV dye.

The experimental CV-dye uptake values were further analyzed using the Freundlich equation, which is applicable for multilayer adsorption on a heterogeneous surface with a non-uniform distribution of heat of adsorption (Fig. 8e). The exponent of the Freundlich model indicates adsorption favorability. In Table 5, the values between 2 and 10 indicate good adsorption characteristics. In this study, the obtained value demonstrates the effectiveness of adsorbents for CV dye ions. Comparing the R² values for both Langmuir and Freundlich models, it was shown that the Langmuir isotherm was the most suitable model to describe the equilibrium data for the adsorption of CV dye onto LSR-F.

The Temkin isotherm model is applied under intermediate sorbate concentration conditions and accounts for a linear decrease in sorption heat logarithmic relationship^41^. The relationship between q_e_ and ln C_e_ is illustrated in Fig. 8f. A high correlation coefficient exceeding 0.97 indicates that the adsorption process based on heat and dye sorption is characterized by a uniform distribution of binding energies.

**Table 4:** Isotherm model parameters for the study of adsorption CV on LSR-F adsorbent (t = 15 min for CV, [dye] = 25 mg/L, dose = 0.05 g, pH = 7, T = 25^◦^C).

Langmuir		Freundlich		Temkin	
q_m_ (mg/g)	73.5343	n_f_ (L/mg)	3.36587	B_t_ (J/mol)	231.456
K_L_ (L/mg)	0.23785	K_f_ (L/mg)	17.5657	K_t_ (L/mg)	1.07949
R^2^	0.99842	R^2^	0.85802	R^2^	0.97287
R_L_	0.01654				

**Fig. 9:**
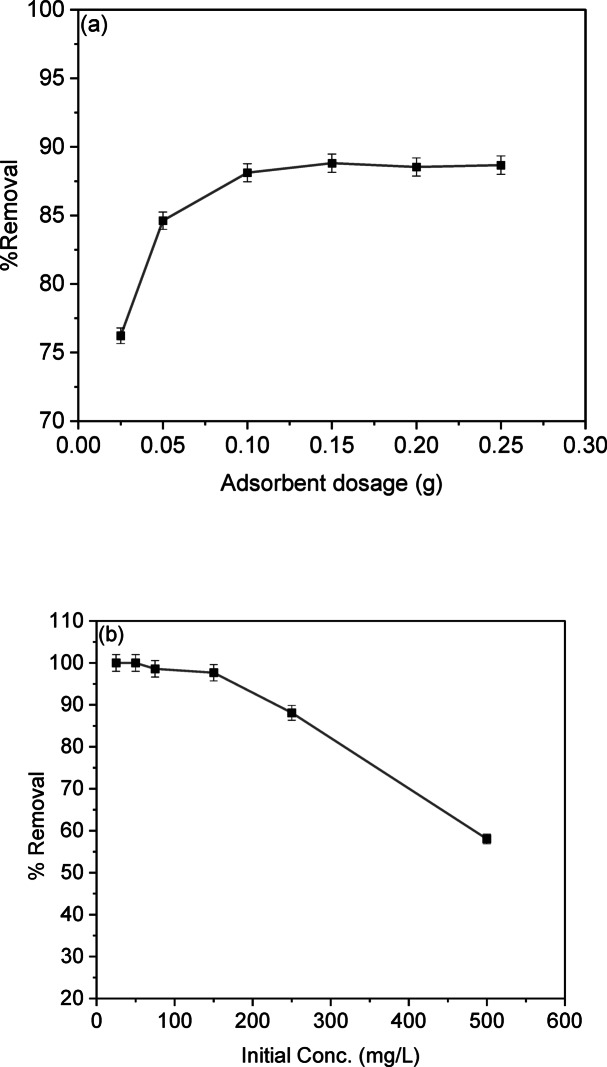

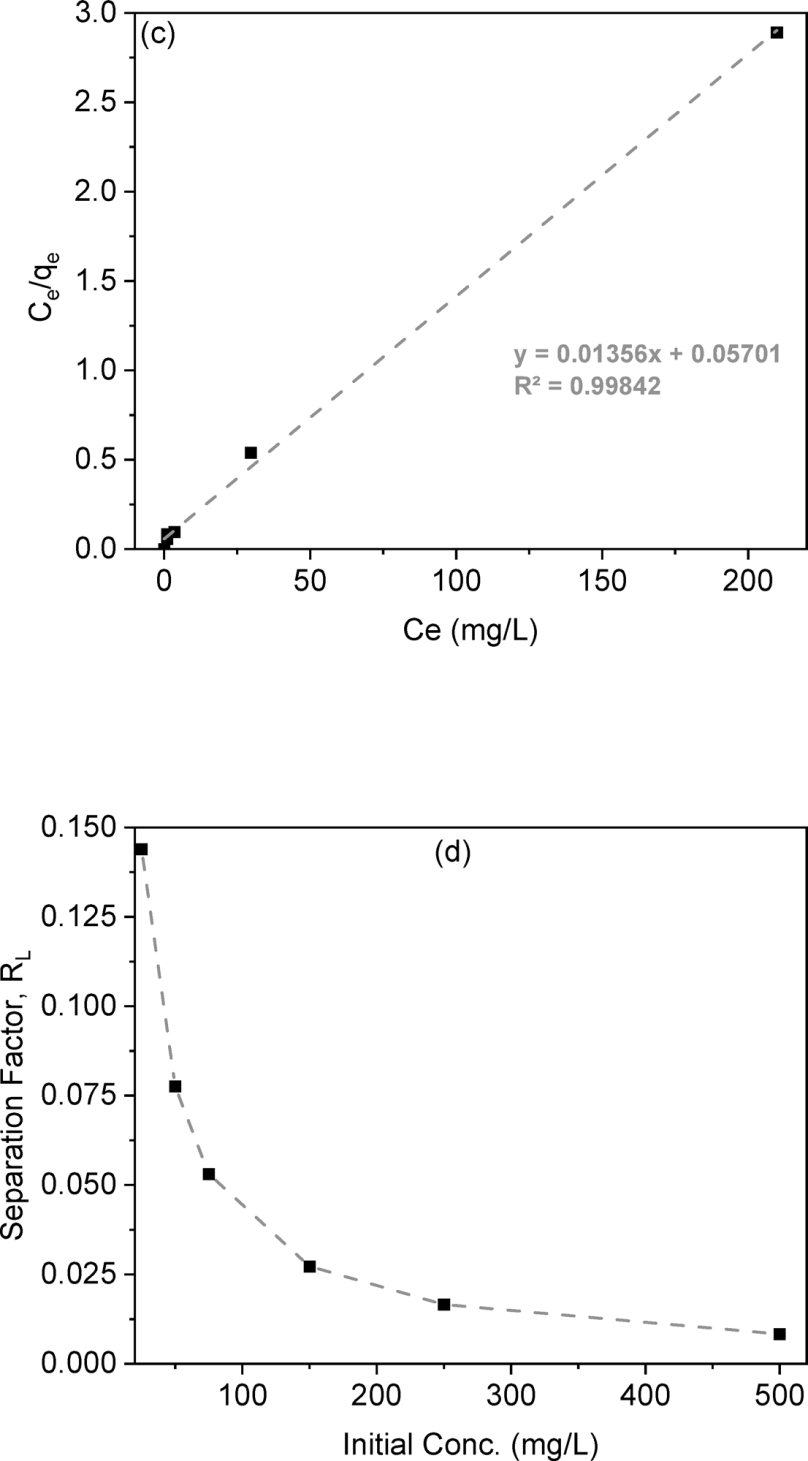

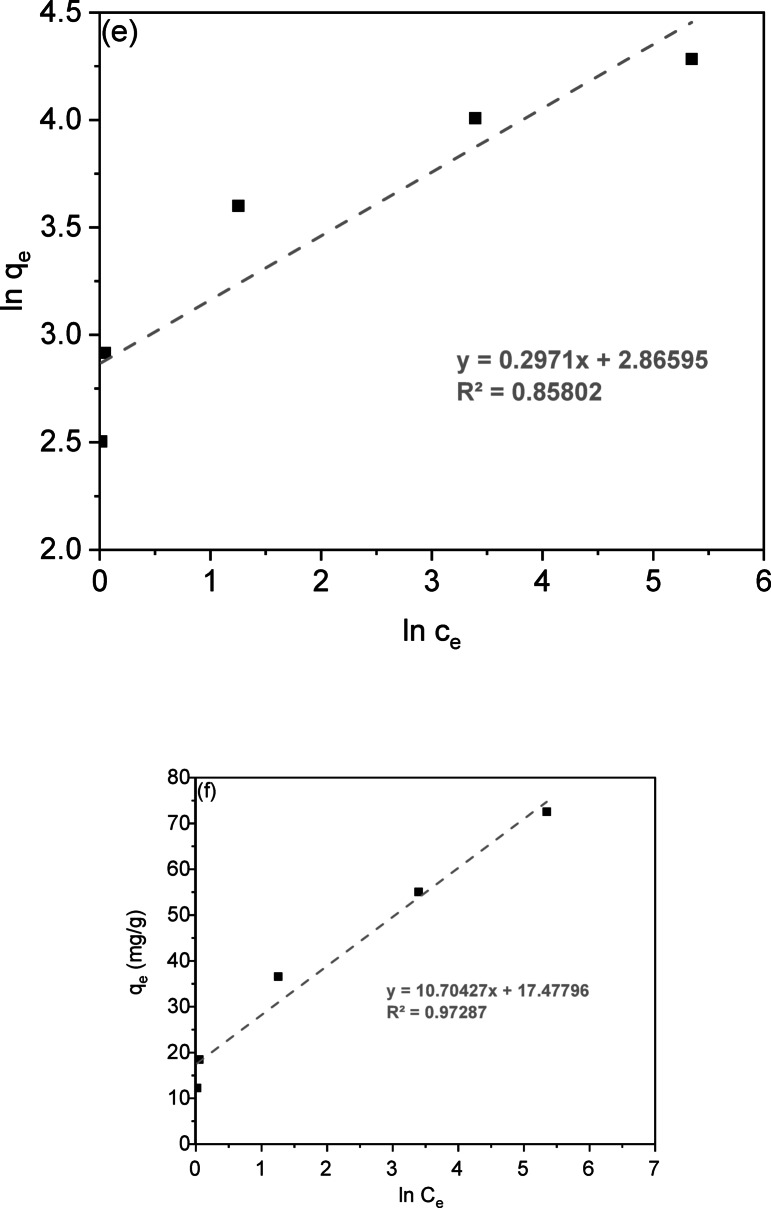
**(a)** Effect of adsorbent dosage on CV removal percentage (t = 15 min for CV, [dye] = 250 mg/L, dose = 0.025, 0.05, 0.1, 0.15, 0.2, and 0.25 g, pH = 7, T = 25 ◦C); **(b)** Effect of the initial dye concentration on the adsorption percentage of CV dye (t = 15 min for CV, dose = 0.1 g, pH = 8, T = 25 ◦C); **(c)** Langmuir isotherm model; **(d)** Separation factor (RL) vs. initial concentration; **(e)** Freundlich; and **(f)** Temkin isotherm model (t = 15 min for CV, [dye] = 25 mg/L, dose = 0.05 g, pH = 7, T = 25◦C).

***Effect of pH;*** The solution’s pH is essential for regulating CV adsorption onto LSR-F adsorbent. It affects the adsorption of H⁺, OH⁻, and other ions, as well as the ionization of CV and the dissociation of functional groups on the adsorbent surfaces^4^. This study examines the effect of solution pH on CV adsorption, conducted within a range of 2 to 12. Results in Fig. 9a illustrate that acidic conditions lower CV removal due to competition between H⁺ ions and CV cations. The maximum CV removal of 89.8% was achieved using LSR-F at a pH of 8. The low pKa value of CV (0.8) facilitates its complete ionization at almost all pH levels, primarily resulting in cationic forms in the solution. Thus, increasing the solution pH above the pKa enhances effective dye removal. Raising the pH from 2 to 8 enhances electrostatic interactions between the negative charges on L, LS, and LSR-F particles and CV dye ions. However, increasing the solution pH to 12 reduces CV removal to 77.6%. This reduction can be attributed to the excessive deprotonation of surface functional groups, which generates a high density of negative charges, combined with the abundance of OH⁻ ions in solution. These factors create strong electrostatic repulsion, ultimately limiting CV adsorption^42^.

### pH-dependent formaldehyde leaching and chemical stability

Leaching studies systematically evaluated formaldehyde (HCHO) release from LSR-F across pH 2–12 (0.10 g LSR-F/100 mL DI water, 24 h, 25 °C) using validated HPLC-DNPH derivatization (C18 column, UV@365 nm; LOD: 0.1 mg/L, R² = 0.999). According to Fig. 9a, the results revealed pH-dependant leaching with minimum release at pH 6 (12 mg/L) governed by hydrolysis equilibrium of resorcinol-formaldehyde network functionalities (Fig. 9a and Fig.S1). Acidic conditions (pH 2–4) promoted maximum release (153–159 mg/L) through proton-catalyzed acetal hydrolysis of –CH₂OH pendants and methylene bridges (R–CH₂–OR → HCHO + R–OH), while near neutral at pH 6 minimized leaching (12 mg/L) via electrostatic stabilization of the crosslinked network. At pH 8, moderate release (94 mg/L) resulted from partial deprotonation of phenolic –OH groups facilitating –CH₂OH equilibrium, whereas alkaline pH 10 (158 mg/L) mirrored acid hydrolysis through OH⁻ nucleophilic attack on methylene bridges, moderated at pH 12 (44 mg/L) by phenolic deprotonation and intramolecular H-bonding stabilization. Total HCHO leaching represented ≤ 0.09% is of incorporated formaldehyde (1.2 mmol synthesis input), confirming excellent crosslinking stability of the resorcinol-formaldehyde network.

### Role of ionic strength, point of zero charge, and zeta potential

As shown in Fig. 9b, in the absence of added salts, the adsorption efficiency reached 95% within 15 min at an initial CV concentration of 40 mg/L and LSR-F dosage of 2 g/L and agitating at 200 rpm for 1 h, followed by filtration of the adsorbent, the dye concentration was measured using UV/visible spectroscopy. The effect of ionic strength on CV dye removal by LSR-F was then investigated by introducing different concentrations (0.1, 0.5, and 1.0 M) of various salts (CaCO₃, CaCl₂, NaCl, LiCl, and ZnCl₂) under the same experimental conditions used in the salt-free system. The presence of inorganic salts significantly altered dye removal. Among the tested salts, CaCO₃ yielded the highest enhancement, achieving approximately 98% removal under optimal conditions. This improvement is likely related to the role of Ca²⁺ ions in modifying electrostatic interactions at the adsorbent surface and potentially inducing localized precipitation or bridging effects that facilitate CV attachment. Generally, the introduction of salts compresses the electrical double layer around both dye molecules and the adsorbent surface, thereby reducing electrostatic repulsion and enhancing adsorption. Additionally, salts can promote the aggregation of CV molecules through charge screening, which reduces their effective solubility and increases hydrophobic interactions with the adsorbent. The salting-out effect may further contribute, as hydrated ions compete strongly for water molecules, lowering dye solubility and favoring transfer to the solid phase. Moreover, the formation of structured ionic atmospheres around charged species can stabilize dye–adsorbent interactions by minimizing Coulombic repulsion. Collectively, these mechanisms explain the observed increase in CV adsorption in the presence of salts, particularly those containing multivalent cations such as Ca²⁺ and Zn²⁺^44^.

To determine the surface charge, the point of zero charge (pHpzc) of the adsorbent surface was examined. The results, as illustrated in Fig. 9c, show a pH_pzc_ value of 7.95 for LSR-F. This means that at pH levels below this value, the positively charged surface of the adsorbents predominates due to equal L − OH₂⁺ sites across this pH range, while at pH levels above this value, the negatively charged surface of the adsorbents predominates due to equal L − S(= O)₂−O⁻ sites across this pH range^44^. The negatively charged surfaces of adsorbents attract positively charged CV dye species through electrostatic interaction at higher pH values (pH > pH_pzc_), thereby improving their adsorption capacity. Additionally, to confirm the interaction mechanism between CV dye and these adsorbents, zeta potential analysis was employed to determine the values of pH at the point of zero charge (pH_pzc_) of the adsorbents^44^.

The zeta potential (|ζ|) method was employed to evaluate the surface charge characteristics of the adsorbent and the associated electrostatic interactions between particles. High absolute zeta potential values (typically |ζ| ≥ 30 mV) indicate strong electrostatic repulsion, which prevents aggregation and ensures colloidal stability. Conversely, at low |ζ| values (< 20 mV), electrostatic repulsion is insufficient to counteract van der Waals attraction, leading to particle aggregation, an increase in hydrodynamic diameter, and a reduction in the accessible surface area for adsorption^45^. For LSR-F, the measured zeta potential was − 32.9 mV, indicating a stable dispersion with strong surface negativity. This negative charge arises from the ionization of oxygen-containing groups in lignin (–OH, –O⁻, =O, –COOH) and the sulfonic groups (–SO₃²⁻, –HSO₃⁻) introduced during sulfonation with Na₂S₂O₃. The measured hydrodynamic diameter of LSR-F was 650.5 nm, suggesting that despite its relatively large particle size, the strong negative surface charge imparted by sulfonation and resorcinol–formaldehyde modification enhances colloidal stability and provides favorable conditions for the adsorption of cationic pollutants such as CV^47^.

### Temperature and thermodynamic study

**Fig. 10:**
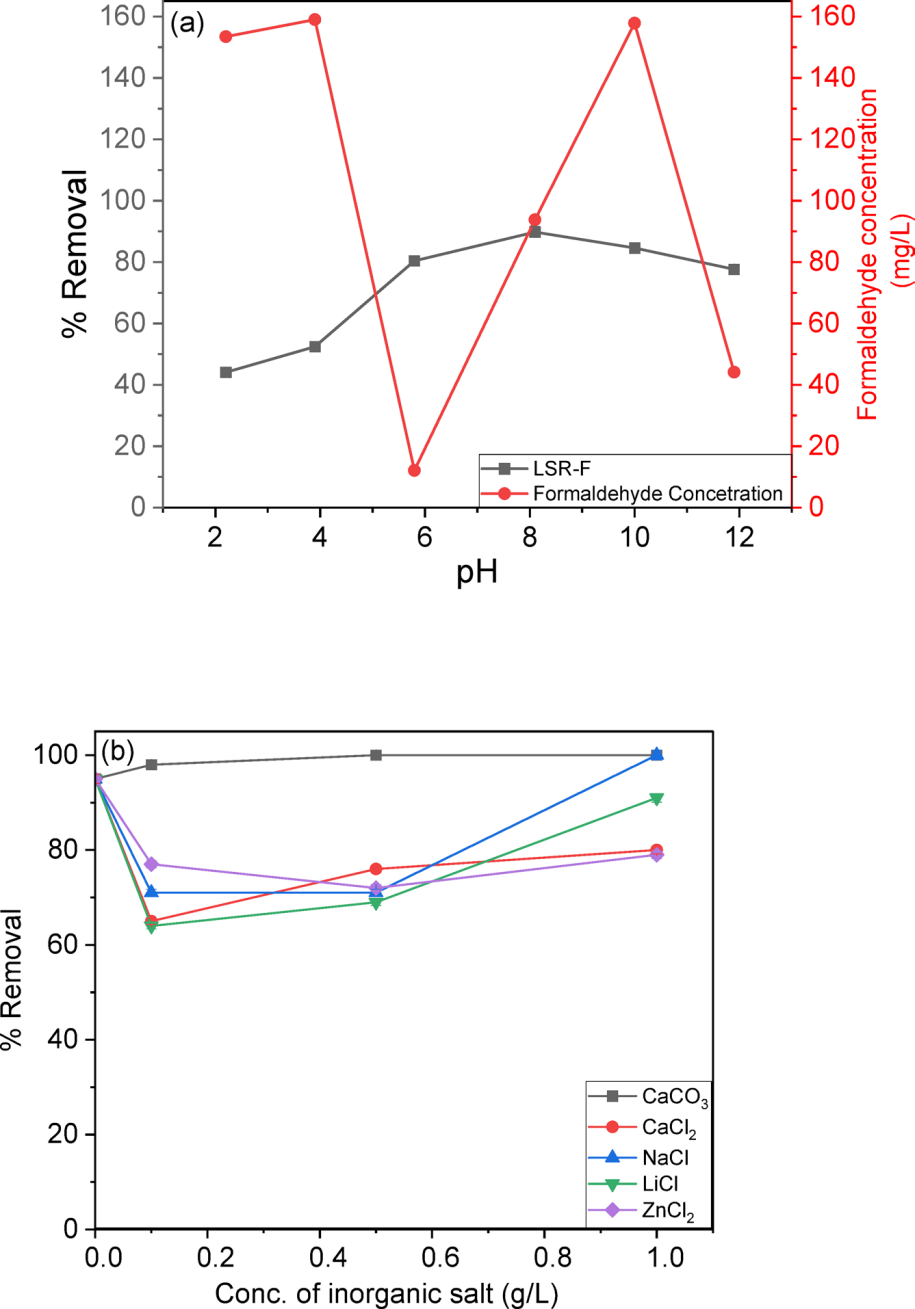

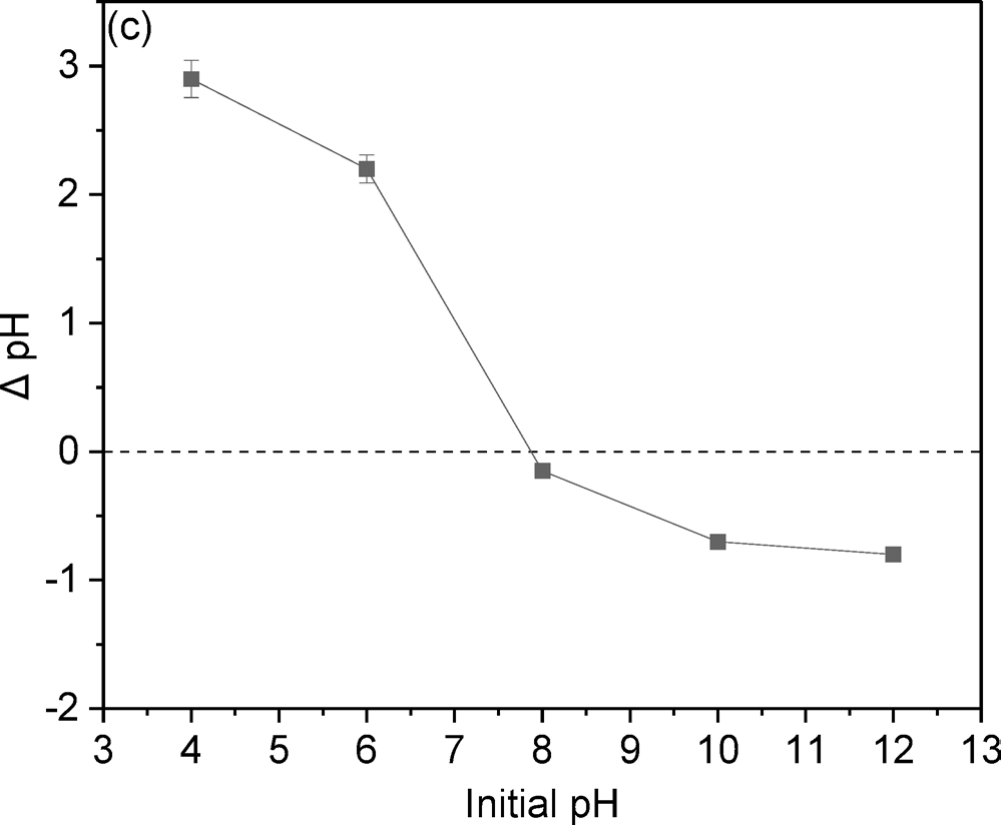
**(a)** Effect of pH on the removal percentage of CV dye from aqueous solution onto LSR-F (t = 15 min for CV, [dye] = 250 mg/L, dose = 0.1 g, pH = 2–12, T = 25 °C); **(b)** Ionic strength of LSR-F adsorbent using CaCO₃, CaCl₂, NaCl, LiCl, and ZnCl₂ salts; and** (c)** Point of zero charge (pHpzc) of the LSR-F, determined by the pH drift method.

*The* effect of temperature on the percentage removal of CV dye onto LSR-F was investigated using 0.1 g of LSR-F adsorbents in a 250 mg/L CV aqueous solution for a 15-minute adsorption process with an agitation speed of 200 rpm, by varying the temperature from 25 to 75 °C, as shown in Fig. 10a. The removal efficiency of CV increased by approximately 5% as the temperature rose from 25 to 75 °C. This characteristic indicates that the CV dye adsorption is an endothermic process. Hence, increasing the solution temperature leads to an increase in the number of active sites available for adsorption^48^. Additionally, it is suggested that the adsorption mechanism of CV dye onto LSR-F adsorbents is considered a chemical adsorption process.

Activation energy is the minimum energy required for a reaction and indicates the energy barrier for adsorbate ions to reach adsorption sites. To assess CV-dye adsorption on LSR-F adsorbent, pseudo-second-order rate constants are employed. The relationship between the rate constant and temperature is described in supplementary information S8. The activation energy provides insight into the adsorption mechanism. To determine the activation energy (Ea) of the process, a plot of ln k₂ versus 1/T was created, as shown in Fig. 10b. The calculated Ea value for CV dye adsorption on LSR-F was approximately 13.68 kJ/mol. Since this value is relatively low (< 40 kJ/mol), it indicates that the adsorption is predominantly governed by weak interactions such as electrostatic attraction, hydrogen bonding, or van der Waals forces. Therefore, the process can be classified as physical adsorption, although the possibility of a minor contribution from activated chemisorption cannot be excluded^12^.

Key thermodynamic parameters include changes in Gibbs free energy (∆*G*°), standard enthalpy (∆*H*°), and standard entropy (∆*S*°) when transferring one mole of solute to the solid-liquid interface. The Van’t Hoff equation describes the relationship between temperature and the adsorption equilibrium constant, as illustrated in the supplementary information (S8). The van’t Hoff plot of ln vs. 1/, illustrated in Fig. 10b, shows a linear relationship with a good correlation coefficient (R²) for different CV-dye concentrations. The slope and intercept allow for the calculation of ∆H° and ∆^∘^, from which ∆G° can be derived in the supplementary information. Table 5 shows ∆H°, ∆S°, and ∆G° values for CV-dye adsorption on L, LS, and LSR-F at different temperatures.

After conducting a thermodynamic analysis, it was found that the treatment applied to the lignin had a significant impact on its behavior. The Gibbs free energy change (ΔG°) was negative, indicating that the reaction is spontaneous under the given conditions and does not require external energy to proceed. In contrast, entropy (ΔS°) has a positive value, reflecting a greater degree of molecular disorder^4^. However, LSR-F displayed a lower Gibbs free energy value, indicating that the reaction is more favorable and spontaneous. ΔG° can decrease with temperature, making the reaction more thermodynamically favorable. As the temperature increases, the mobility of dye molecules increases, causing the dye to escape from the solid phase to the liquid phase. As listed in Table 5, it demonstrated that the adsorption process became more favorable at higher temperatures, indicating that thermal energy supports the progression of the reaction. Additionally, a positive ΔH° indicates an endothermic process and strong interaction between the adsorbent and adsorbate^49^.

**(b)** The Van’t Hoff plot at 0.1 g concentration and pH 7 examines thermodynamic parameters across different temperatures.

**Table 5:** Thermodynamic parameters for the adsorption of CV-dye ions onto LSR-F.

Adsorbent	ΔH (kJ/mol)	ΔS (J/K·mol)	ΔG (kJ/mol)				
			25 °C	35 °C	45 °C	55 °C	65 °C
LSR-F	11.722	44.167	−1.439	−1.881	−2.323	−2.764	−3.206

### Adsorption mechanism

There are two major types of mechanisms between LSR-F (adsorbent) and CV dye molecules (adsorbate): physical and chemical adsorption. The experimental findings align well with the pseudo-second-order kinetic model and the intra-particle diffusion kinetic models. According to kinetic studies, the electrostatic interaction of the cations controls the adsorption process in the initial stage. As the system approaches equilibrium, intra-particle diffusion limits CV adsorption by LSR-F. Because the adsorption process is quite complicated and may include several mechanisms, it is critical to understand the physical and chemical adsorption mechanisms as follows:

### Physical mechanism

**Fig. 11:**
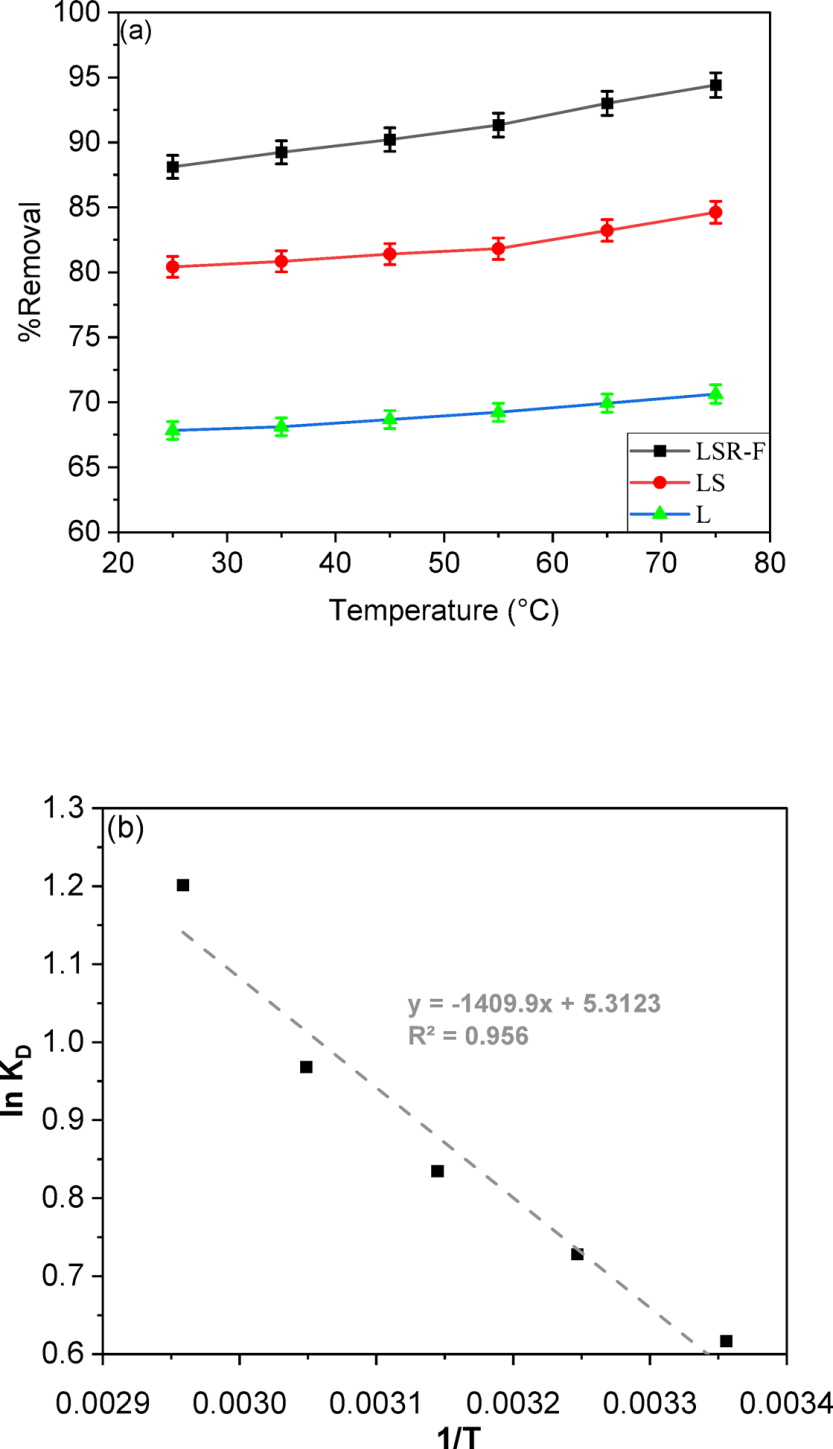
**(a)** Effect of temperature on the percentage removal of CV dye at T = (25, 35, 45, 55, 65, 75 °C), t = 15 min for CV, [dye] = 250 ppm, adsorbent dose = 0.1 g, pH = 7), **(b)** The Van’t Hoff plot at 0.1 g concentration and pH 7 examines thermodynamic parameters across different temperatures.

The adsorption of CV dye onto the LSR-F adsorbent involves a multifaceted mechanism, as illustrated in Fig. 11a, comprising several mass transfer and diffusion steps. According to the results from the intra-particle diffusion model, initially, CV dye molecules migrate from the bulk solution to the external surface of the LSR-F particles through film diffusion. Subsequently, intra-particle diffusion governs the penetration of dye molecules into the pores and capillaries of the material, enabling access to internal adsorption sites. Finally, once on the surface, CV molecules may diffuse laterally to locate and occupy available sites, a process referred to as surface diffusion^50^.

### Chemical mechanism

The adsorption mechanism was thoroughly investigated, showing that its effectiveness depends on both the structural characteristics of the adsorbate molecules and the surface chemistry of the LSR-F adsorbent. The mechanisms for CV dye removal by LSR-F are illustrated in Fig. 11b. CV is a cationic, water-soluble dye, whereas LSR-F contains aromatic structures with oxygenated and sulfonated functional groups, making it amphiphilic. This dual nature allows LSR-F to interact with CV through multiple mechanisms, including π–π stacking, electrostatic attraction, van der Waals forces, hydrogen bonding, and hydrophobic interactions.

**Fig. 12:**
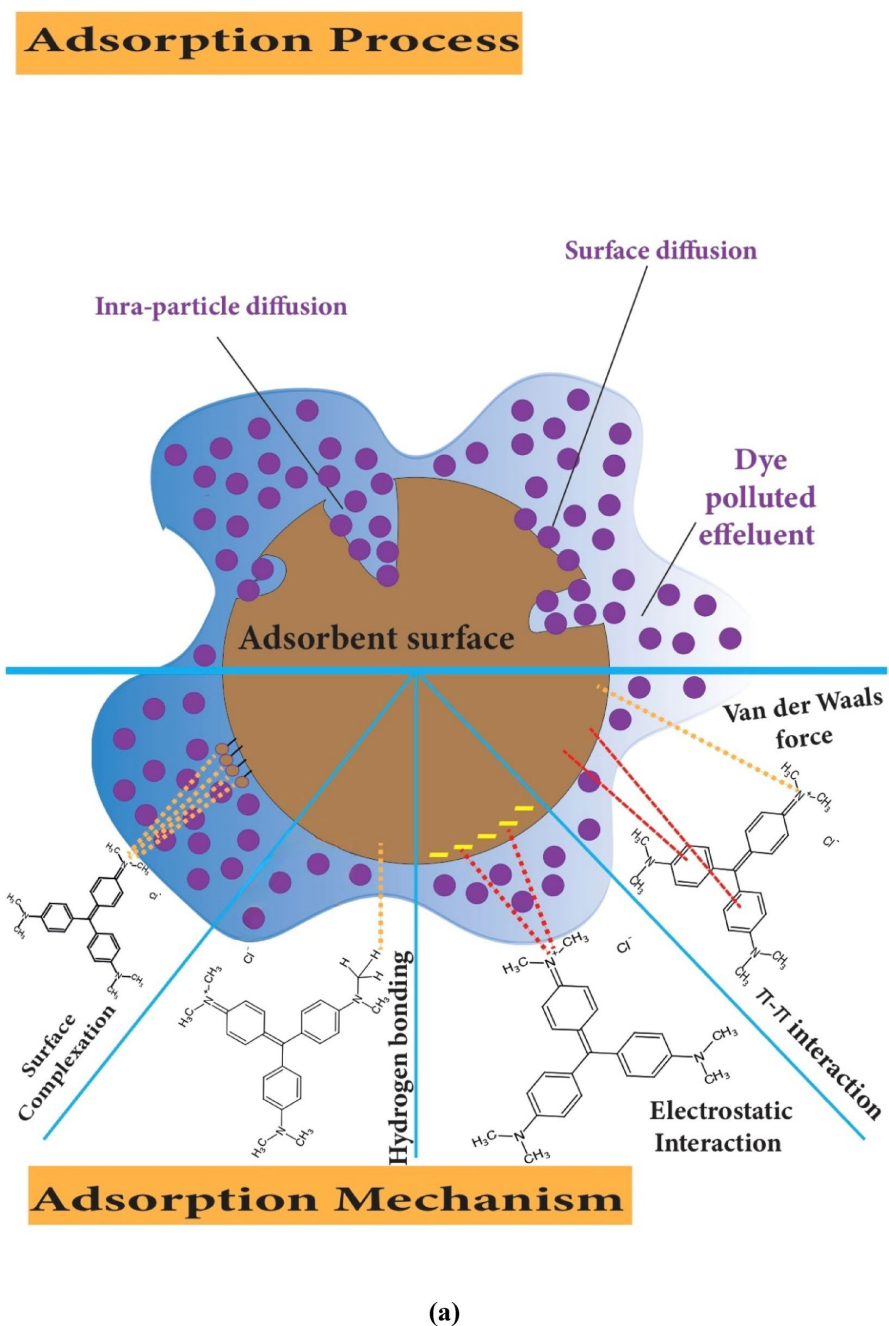

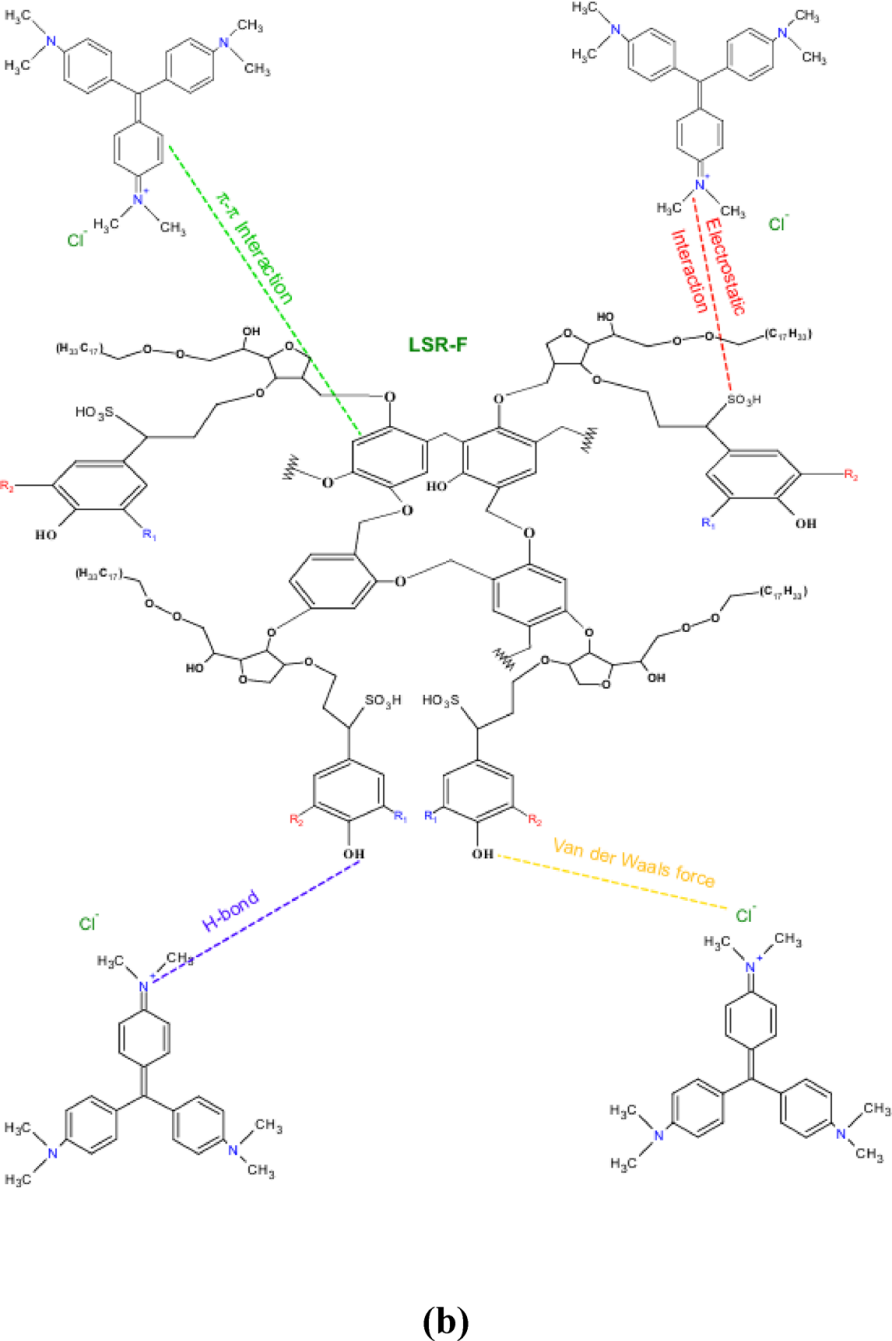
**(a)** Schematic diagram of the adsorption mechanism for the adsorption of CV onto LSR-F; and **(b)** chemical mechanism of the adsorption.

The FT-IR analysis of the LSR-F indicated a number of oxygenated functional groups, such as hydroxyl, carboxylic, phenolic, carbonyl, and ether groups, which likely play a role in the adsorption of CV dye, as shown in Fig. 12. The aromatic domains of LSR-F provide hydrophobic sites that stabilize π–π and hydrophobic interactions with the dye’s aromatic rings, while polar groups (–OH, –COOH, –SO₃⁻) facilitate hydrogen bonding and electrostatic interactions with the iminium groups of CV. These various interactions align with the assumption of the adsorption heterogeneity, suggesting the Langmuir and Temkin isotherm models. Overall, the adsorption capacity of lignocellulosic-based materials like LSR-F arises from the synergy of weak intermolecular forces, surface charge effects, and the amphiphilic nature of the adsorbent^51^. The adsorption of CV dye onto LSR-F involves several complementary interactions. Firstly, van der Waals forces provide a baseline contribution to adsorption through weak, nonspecific attractions between CV molecules and the LSR-F surface; however, these interactions alone are insufficient to explain the high adsorption efficiency. Secondly, π–π interactions play a major role, as LSR-F’s aromatic structures enable stacking with the conjugated aromatic rings of CV. In this interaction, the cationic π-system of CV acts as an electron acceptor, while the electron-rich π-domains of LSR-F act as donors, stabilizing the adsorbate–adsorbent complex. Thirdly, hydrogen bonding contributes significantly, since oxygen-containing groups on LSR-F (–OH, –C = O, –SO₃⁻) can act as hydrogen bond donors or acceptors with the amino and iminium groups of CV. Finally, electrostatic attraction is a key mechanism: CV carries a permanent positive charge (–N⁺=), while LSR-F develops negatively charged oxygen- and sulfur-containing groups under neutral to basic conditions. This charge complementarity facilitates strong Lewis acid–base interactions, further enhancing dye adsorption^52^.

### Desorption and recycling study

**Fig. 13:**
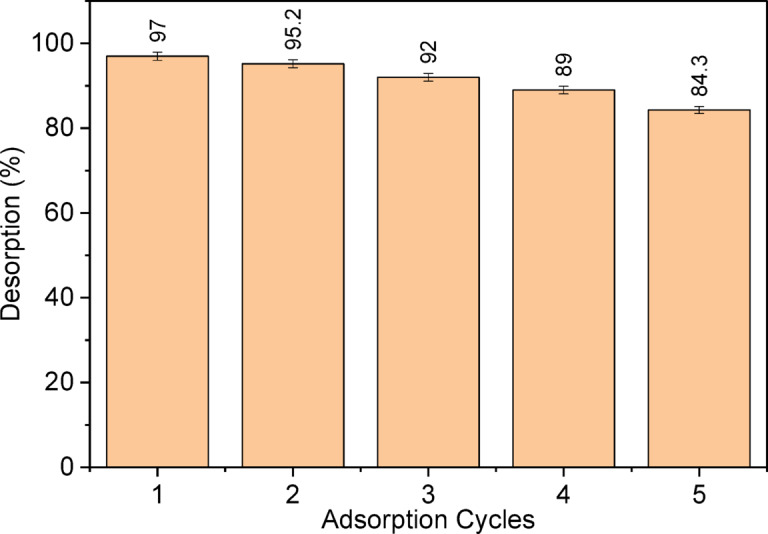
Desorption and recycling of LSR-F adsorbent using 0.1 M HCl.

A key economic factor in material selection is its reusability. Studies show that reusing acids as desorbing agents effectively removes cationic dye^53^. Figure 13 illustrates that 0.1 M HCl significantly enhances the resorption process. The LSR-F adsorbent retains 99% removal efficiency initially, dropping to 84.3% after five cycles due to hydrogen ions competing with dye molecules for adsorption. Based on the findings above, the LSR-F adsorbent is cost-effective and suitable for industrial applications, recommended for continuous systems with a minimal residual time to maximize its effectiveness.

Role of LSR-F as an adsorbent for the removal of CV dye in advancing Sustainable Development Goals (SDGs).

Industrial wastewater contaminated with hazardous dyes, such as crystal violet (CV), threatens the integrity of ecosystems and undermines the quality of water resources essential for drinking, agriculture, and aquatic life. This pollution directly challenges several Sustainable Development Goals (SDGs). Most notably, it hinders SDG 6: Clean Water and Sanitation, which seeks to ensure universal access to safe and sustainably managed water resources. Additionally, untreated dye effluents jeopardize aquatic ecosystems, reducing biodiversity and thereby undermining SDG 14: Life Below Water, which emphasizes the conservation and sustainable use of marine and freshwater environments^54^.

In addressing these challenges, LSR-F, a lignin-based sulfonated resorcinol–formaldehyde adsorbent synthesized from agricultural biomass, offers a sustainable and effective solution for CV dye removal. Its use exemplifies the valorization of agricultural residues, supporting SDG 12: Responsible Consumption and Production by reducing dependency on virgin raw materials and promoting circular economy practices. Furthermore, substituting conventional waste disposal with the production of value-added adsorbents like LSR-F contributes to SDG 13: Climate Action, as it mitigates pollution while lowering greenhouse gas emissions linked to open burning or landfilling of agricultural residues^55^^56^.

The sustainability of LSR-F can be further validated using Life Cycle Assessment **(LCA)**, which evaluates the environmental impacts associated with its production and application. This approach underscores its potential to minimize ecological harm, optimize resource efficiency, and reinforce the integration of waste-to-resource technologies. Collectively, the application of LSR-F not only advances water purification technologies. but also demonstrates a holistic contribution to achieving multiple SDGs through sustainable environmental management and innovation^57^.

### Comparison of maximum adsorption capacities with different types of lignin-based adsorbents

According to the Langmuir model, the maximum multilayer adsorption capacity of CV ions on the LSR-F adsorbent prepared was determined to be 73.53 mg/g. This value aligns closely with the maximum theoretical adsorption capacity of CV ions as previously reported. To assess the efficiency of the LSR-F adsorbent in removing CV ions compared to other adsorbent materials, the maximum obtained adsorption capacities were compared. Tables 6 and 7 illustrate a comparative analysis of the adsorption performance of the prepared LSR-F adsorbent for CV ion removal and the adsorption performance of lignocellulosic adsorbents for different dye ion removal in relation to literature values, respectively, demonstrating an outstanding adsorption capability.

**Table 6:** Comparable investigation of the removal capacity of CV-dye ions onto different types of lignin-based adsorbents.

Adsorbent	Adsorbate	Maximum adsorption capacity (mg/g)	Ref.
Magnetic turned sorghum husk	Crystal Violet dye	18.8	^53^
Oak tree fruit waste (activated carbon (AC))	Crystal Violet dye	23.86	^59^
Almond shells	Crystal Violet dye	12.20	^60^
Activated Carbon of Lemon Wood (ACL)	Crystal Violet dye	23.6	^61^
Nascent Rice Husk	Crystal Violet dye	24.4781	^62^
LSR-F	Crystal Violet dye	73.53	Present study

**Table 7:** Comparable investigation of the removal capacity of different dye ions onto the different types of lignin-based adsorbents.

Adsorbent	Adsorbate	Maximum adsorption capacity (mg/g)	Ref.
Palm Peat (PP)	Methylene blue (MB) dye	21.12	^63^
Paracentrotus lividus shells (PLS)	Malachite green dye	22.35	^64^
Magnetic turned sorghum husk	Methylene blue (MB) dye	30	^53^
Lignin-derived carbon nanosheet	Rhodamine B dye	41.2	^65^
Quaternized lignin	Rhodamine B and methylene blue (MB) dye	41.85, 49.47	^66^
LSR-F	Crystal Violet	73.53	Present study

## Conclusions

An innovative lignin-based adsorbent derived from corn stover (LSR-F) was successfully developed for the efficient removal of crystal violet (CV) dye from aqueous solutions. Rich in functional groups such as hydroxyl and carbonyl moieties, LSR-F demonstrated a strong affinity toward CV molecules. Under optimal conditions (250 mg/L CV, pH 8, room temperature), it achieved an impressive adsorption capacity of 73.53 mg/g within just 15 min of equilibrium. The adsorption followed pseudo-second-order kinetics and was best described by the Langmuir isotherm, confirming a favorable and uniform interaction between the adsorbent and the dye molecules. Importantly, LSR-F exhibited excellent reusability, retaining high removal efficiency across multiple regeneration cycles. These findings highlight LSR-F as a cost-effective, sustainable, and high-performance adsorbent for the treatment of dye-contaminated wastewater, offering significant potential for advancing environmentally friendly water purification technologies.

## Supplementary Information

Below is the link to the electronic supplementary material.

Supplementary Material 1

## Data Availability

All data generated or analyzed during this study are included in this article (and its Supplementary Information file).
