# Supplementary material for: Utilization of wastes from bioethanol production for the fabrication of new adsorbents for the removal of toxic dye in water
Source: Sci Rep. 2026 Jan 27;16:3473. doi: 10.1038/s41598-026-35236-8 (PMC12847803; doi:10.1038/s41598-026-35236-8)
Supplement: Supplementary file 1 — Supplementary Material 1 [file 41598_2026_35236_MOESM1_ESM.docx]

**Supplementary Information**

## **Utilization of wastes from bioethanol production for the fabrication of new adsorbents for the removal of toxic dye in water**

## **Khloud Eltaher ^1^, Sara E. AbdElhafez ^2,*^, Rehab M. Ali ^2,*^, Ayman El-Faham ^1^, Ali A. El‑Bardan ^1^, Hesham Hamad ^2,*^**

^1^ Chemistry Department, Faculty of Science, Alexandria University, Ibrahimia 21321, Alexandria, Egypt.

^2^ Fabrication Technology Research Department, Advanced Technology and New Materials Research Institute (ATNMRI), City of Scientific Research and Technological Applications (SRTA-City), New Borg El-Arab City 21934, Alexandria, Egypt.

Corresponding author: [heshamaterials@hotmail.com](mailto:heshamaterials@hotmail.com) (Hesham Hamad); [sara.elsayedeg@gmail.com](mailto:sara.elsayedeg@gmail.com) (Sara E. AbdElhafez), [rehabmohamedali1983@gmail.com](mailto:rehabmohamedali1983@gmail.com) (Rehab M. Ali)

**S1. Composition (Lignin, Cellulose and Hemicellulose Contents);** Quantitative analysis of the CS contents lignin, cellulose and hemicellulose was performed as mentioned in ^1^, In each trial, a 1 gram portion of the dried CS sample was individually mixed with 70 mL of neutral detergent and subjected to autoclaving at 100°C for 40 minutes, followed by an additional 20 minutes at 120°C. The solid residues obtained were then filtered, rinsed with hot distilled water until the pH reached 6.5–7, washed twice with ethanol, and left to dry overnight at 50°C. These dried residues were weighed and designated as (W_0_). Subsequently, the (W_0_) sample was mixed with 70 mL of 2 mol/l HCl and autoclaved at 100°C for 60 minutes. The resulting solid residue was filtered, rinsed with hot distilled water until the pH reached 6.5–7, washed twice with ethanol, dried overnight at 50°C, and weighed, being labeled as (W_1_). The W_1_ sample underwent immersion in 72% H_2_SO_4_ for 4 hours at room temperature, followed by the addition of water to the mixture, which was then incubated overnight at room temperature. The resulting solid residue was filtered, washed with hot distilled water until reaching a pH of 6.5–7, dried overnight at 50°C, weighed, and labeled as (W_2_). Subsequently, the (W_2_) sample was subjected to combustion in a muffle furnace (Barnstead Thermolyne 48000, Ramsey, MN, USA) at 575°C for 4 hours. The resulting powder was weighed and designated as (W_3_). The content of hemicellulose, cellulose, lignin, and ash was estimated using the following equations ^1^:

Hemicellulose = W_0_ − W_1_ (1)
 Cellulose = W_1_ − W_2_ (2)
 Lignin = W_2_ − W_3_ (3)
 Ash = W_3_ (4)
Following by determination of chemical composition, the % hemicellulose removal, % cellulose removal, and % lignin recovery, which were calculated by Equations (5), (6), and(7):
 % Hemicellulose removal = (H_i_ − (H_f_ × % Solid recovery yield/100)) /H_i_ ) × 100 (5)
The percentage of hemicellulose removal during the treatment process is calculated by comparing the initial hemicellulose content (H_i_) with the final hemicellulose content (H_f_) in the solid residue, expressed as a percentage of the initial hemicellulose content.
 % Cellulose removal = (C_i_ − (C_f_ × % Solid recovery yield/100)) /C_i_ ) × 100 (6)
The percentage of cellulose removal during the treatment process is calculated by comparing the initial cellulose content (C_i_) with the final cellulose content (C_f_) in the solid residue, expressed as a percentage of the initial cellulose content.

% lignin recovery = (L_f_ × % Solid recovery yield/100)) /L_i_ ) × 100 (7)
The percentage of lignin recovery during the treatment process is calculated by comparing the initial lignin content (L_i_) with the final lignin content (L_f_) in the solid residue, expressed as a percentage of the initial lignin content, as specified in the study. The solid recovery yield was determined using Equation (8):
% Solid recovery yield = (Final treated weight (g) / Initial CS weight (g)) × 100 (8)
**S2. Proximate analysis, ultimate analysis, and higher heating value (HHV);** Adsorbents underwent proximate analysis to assess its moisture content (MC), volatile matter (VM), fixed carbon (FC), and ash content. The MC was determined according to the DIN 51718 standard method. Specifically, 1 gram of adsorbents was weighed on dried glass Petri Dishes and heated in a furnace at 105°C for 3 hours^2^. The MC of adsorbents was calculated based on the initial mass of adsorbents before (Mi) and after the heat treatment (Mf).

The weight reduction, which is directly related to f (VM), was determined using the DIN 51720 standard method. This involved subjecting 1 gram of the sample to TGA analysis, where the weight loss (excluding moisture) was measured as the sample was heated to 919°C with a heating rate of 10°C per minute for 7 minutes under an N_2_ atmosphere. Furthermore, the ash content was determined using the DIN 51719 standard method. The sample was returned to the furnace and heated gradually to 750°C for 1 hour, followed by cooling to ambient temperature in a desiccator. The weight of the resulting ash was recorded. These standardized methods were employed to accurately determine the moisture content, volatile matter, and ash content of the adsorbents. Fixed carbon represents the solid fuel component of biomass. Most standards, including ASTM D Standard 3172–73, determine it by subtracting the moisture, ash, and volatile matter from the initial biomass weight using the following formula (Equation 9) ^3^ :

Fixed carbon (%) = 100 - (moisture % + ash % + volatile matter %) (9).
 Adsorbents underwent ultimate analysis using a Vario-Micro CHN elemental analyzer (Elementar Analyses system GmbH, Langenselbold, Germany) to determine the CHNS/O elemental composition of CS. The analysis followed the ASTM E-775-78 standard method, which involved the simultaneous combustion of gases to analyze the elements C, H, N, and S. Additionally, by obtaining the elemental composition, the higher heating value (HHV) can be calculated using the following equation, which is commonly used for engineering calculations ^4^.

HHV = 0.3419 × (%C) + 1.1783 × (%H) − 0.1034 × (%O) + 0.1005 × (%S) − 0.0151 × (%N) − 0.0211 × (% Ash) [MJ/kg] (10)

**S3. Physico-chemical characterizations**; the thermal stability of the CS, L, LS, and LSR-F was investigated through thermogravimetric analysis (TGA) using the Shimadzu TGA-50 instrument from Kyoto, Japan. The analysis was carried out under a nitrogen atmosphere, starting from room temperature and gradually increasing up to 919°C at a heating rate of 10°C per minute. During the TGA, the heating rate, weight loss, moisture content, volatile content, ash content, and fixed carbon content of the tested samples were recorded. A curve depicting weight loss against temperature was constructed from the obtained data. CHNS contents were determined using a Vario-Micro CHN elemental analyzer (Elementar Analyses system GmbH, Langenselbold, Germany). ASTM E-775-78 standard method was utilized to analyze the elements C, H, N, and S during the simultaneous combustion of the gases. Raman analysis was performed using (Senterra II instrument from Bruker, Germany). For FTIR analysis, a FTIR instrument (Shimadzu FTIR-8400 S, Kyoto, Japan) was utilized. The samples were finely ground with 100 mg of KBr to achieve a uniform powder. The resulting mixture was then pressed into pellets using a hydraulic press. Absorption spectra were obtained in the range of 4000 to 500 cm^-1^ with a resolution of 4 cm^-1^. To capture surface morphology images, a scanning electron microscope (SEM) were employed (JEOL Model JSM 6360 LA, Tokyo, Japan).

**S4. Surface charge measurement.** The surface charges of L, LS, and LSR-F adsorbents were analyzed using a Zetasizer Nano ZS (Malvern Instruments, Malvern, UK). Electrophoretic mobility was measured by dispersing the adsorbents in deionized water, and zeta potential was measured at reflux index (RI) =1.33 and calculated with the Smulochowski approximation. The pH_pzc_ for these adsorbents was carried out using the salt addition method with five 0.1 M NaCl solutions at pH 2 to 12 by adjusting the pH using 0.1 M NaOH and 0.1 M HCl. A quantity of 0.25 g of adsorbents was mixed separately with 25 ml of solution, shaken at 200 rpm for 24 hours at room temperature. The final pH values were measured to estimate pH_pzc_ by plotting the differences between initial and final pH against initial pH values

**S5. Adsorption study;** A standard solution of crystal violet (CV) dye at a concentration of 500 ppm was prepared as the initial solution. Subsequent concentrations were obtained by diluting the standard solution. Batch experiments were conducted in a 50 mL Erlenmeyer flask placed on an orbital shaker operating at 200 rpm and maintained at room temperature until equilibrium was attained. A fixed quantity of prepared adsorbents L, LS, and LSR-F was mixed with 25 mL of the dye solution. At different time intervals, samples were collected from the reaction media using a filter syringe, and the residual CV dye concentrations were determined using a UV/visible spectrophotometer (model 7230 G, manufactured in Shanghai, China) at the maximum absorbance wavelength of 570 nm. This measurement step was repeated three times to ensure accuracy. The effects of various parameters, including contact time (ranging from 1 min to 180 min), pH levels (2, 4, 6, 8, 10 and 12), adsorbate solution concentrations (25, 50, 75, 150, 250 and 500 ppm), adsorbent doses ranging from (0.025 to 0.25 g), and temperatures (25, 35, 45, 55, 65 and 75 °C), were investigated to determine the optimal conditions. The CV concentration of the filtrate and the removal efficiency were determined after each cycle using ultraviolet spectrophotometer. The equilibrium adsorption capacity of CV, q_e_ (mg g^−1^ ), was calculated according to equations;

$q_{e (adsorp.)=} \frac{(C_{0}-C_{e} )V}{W} (11)$
 Where q_e_ (mg L^−1^) is the initial concentration of CV solution, (mg L^−1^) is the equilibrium concentration of the CV solution after adsorption, V (L) is the volume of CV solution, and W (g) is the quantity of adsorbent used.

**S6. Kinetic Studies****;** Kinetic models play a crucial role in understanding the mechanism and rate-limiting steps involved in the adsorption process. In this study, the kinetics of CV adsorption onto the L, LS, and LSR-F surfaces were investigated by fitting the experimental data to several models: pseudo-first-order, pseudo-second-order, intraparticle diffusion, Boyed, and the Elovich models. The pseudo-first-order model primarily considers the diffusion and mass transfer of CV onto the L, LS, and LSR-F surfaces. On the other hand, the pseudo-second-order model suggests that chemisorption is the rate-limiting step in the adsorption process. The intraparticle diffusion model helps in understanding the potential mechanisms of adsorption for the studied molecules and verifies the transport mechanism involved. Lastly, the Elovich model assumes that the solid surface is energetically heterogeneous, which can influence the adsorption process.

**S7. Isothermal studies;** they conducted to examine the adsorption behavior of CV at room temperature and various initial concentrations (25-500 ppm). A fixed amount of adsorbent weighing 0.1 g was added to a 25 mL CV solution and subjected to constant shaking at 200 rpm. The Langmuir, Freundlich, and Temkin isotherm models were employed to analyze the adsorption process. The Langmuir, Freundlich, and Temkin models were studied to assess the interaction between CV and LSR-F and determine the adsorption capacity of LSR-F. These models provided insights into whether the adsorption occurred in a monolayer or multilayer fashion ^5,6^.

**S8. *Estimation of thermodynamic parameters.***

The thermodynamic parameters are the standard free energy change (*ΔG°*), the standard enthalpy change (*ΔH°*), and the standard entropy change (*ΔS°*) for the adsorption of CV dye onto LSR-F nanocomposites at various temperatures were calculated and listed Table 5. The apparent thermodynamic parameters, ΔH^°^ and ΔS^°^, for CV adsorption using synthesized LSR-F are calculated from the slopes and intercepts of the linear variation of ln K_c_ vs. 1/T as shown in Fig. 11 (b) by using the following equation:

ln K_c_ = (ΔS^°^/ R) – (ΔH^°^/RT) (12)

Where R is the universal gas constant, 8.314 J mol^−1^ K^−1^ and T is the absolute temperature in Kelvin. The distribution coefficient K_c_ was calculated using the following equation:

K_c_ = $\frac{q_{e}}{C_{e}}$ (13)

The ΔG^°^ for the adsorption process is calculated by using the following equation:

ΔG^°^ = ΔH^°^ − TΔS^°^ (14)

The relationship between the rate constant and temperature may be described by the linear form of the Arrhenius equation that expressed as follows

ln*k*_2_ = ln*A* −*E*_a_ / *RT* (15)

where *E*_a_is the activation energy, J / mol, *A* is the Arrhenius constant, g / mg min, *k*_2_is thepseudo-second-order rate constant of adsorption, g / mg min(Table 3), *R* is the ideal gas constant, 8.314 J / mol K, and *T* is the temperature of the solution, K.

**S9. Ionic strength;** The impact of ionic strength, resulting from the presence of other salts, on the removal efficiency of crystal violet (CV) using L, LS, and LSR-F adsorbents was investigated through the following procedure. Solutions of three different concentrations (0.1, 0.5, and 1 M) of five different salts, namely CaCO_3_, ZnCl_2_, CaCl_2_, NaCl, and LiCl, were prepared by dissolving a specific amount of each salt in 25 mL of a CV solution with an initial concentration of 10 ppm. A mass of 0.05 g of L, LS, and LSR-F were added to the prepared solution, followed by agitation for 1 hr. at 200 rpm. Subsequently, the adsorbent was separated by filtration, and the concentration of the dye in the liquid phase was determined using a UV/visible spectrophotometer.

**S10.** **Determination of formaldehyde in wastewater through sodium sulfite method**

In the sodium sulfite method (Hantzsch Reaction – Indirect Titration) for the detection of formaldehyde in wastewater, a known volume of the sample is transferred into a conical flask and, if necessary, diluted with distilled water for accurate titration. An excess of 10% (w/v) sodium sulfite solution is then added, and the mixture is stirred and allowed to react at room temperature for 5–10 minutes. During this reaction, formaldehyde combines with sodium sulfite to form hydroxymethanesulfonate, releasing sodium hydroxide (NaOH) in the process. After the reaction is complete, 2–3 drops of phenolphthalein indicator are added, producing a pink color due to the presence of NaOH. The liberated NaOH is then titrated with a standard hydrochloric acid (HCl) solution until the pink color just disappears, indicating neutralization. From the volume of HCl used, the amount of formaldehyde present in the wastewater sample can be determined.

**Table S1:** **Kinetic models utilized to describe CV dye adsorption process onto LSR-F.**

| Model | Equation | Abbreviations |
| --- | --- | --- |
| Pseudo-first order | $\ln\left( q_{e} - q_{t} \right)=\ln q_{e}- k_{1}t$ | where $q_{e}$ and $q_{t}$represent the amounts of CV-dye adsorbed (mg/g) at equilibrium and at time $t$ (min), respectively, and $k_{1}$ is the pseudo first-order adsorption rate constant (min^-1^) |
| Pseudo-second order | $\frac{t}{qt}=\frac{1}{\left( k_{2}*{q_{e}}^{2} \right)}+\frac{t}{q_{e}}$ | where $k_{2}$represents the equilibrium rate constant of pseudo-second order (g/mg min), $q_{e}$ signifies the amount  of adsorption at equilibrium, and $q_{e}$_(cal.)_ denotes the calculated equilibrium adsorption. |
| Intraparticle diffusion | $q_{t}=a+ k_{int}.t^{0.5}$ | where $k_{int}$ represents the intra-particle diffusion rate  constant (mg/g min), and a is an intercept indicating the thickness of the boundary layer. |
| Boyd | $B_{t}= -0.4977-\ln\left( 1-F \right)$ | where $F$ represents the fraction of solute adsorbed at time t (minutes), calculated from  $F= q_{t}/q_{e}$ , q_e_ is the amount of solute sorbed (mg/g) at infinite time. |
| Elovich | $q_{t}=\frac{1}{\beta_{e}}\ln\left( \alpha\beta_{e} \right)-\frac{1}{\beta_{e}}\mathrm{lnt}$ | where α represents the initial rate (mg/g min)  as $q_{t}$approaches zero, while β relates to activation energy  and surface coverage for chemisorption (g/mg). |

**Table S2: Isotherm models utilized to describe CV dye adsorption process onto LSR-F.**

| Model | Equation | Abbreviations |
| --- | --- | --- |
| Langmuir | $\frac{C_{e}}{q_{e}}=\frac{1}{q_{m}K_{L}} +\frac{C_{e}}{q_{m}}$ | where q_e_ is the adsorption capacity at  equilibrium (mg/g), q_m_ is the maximum adsorption capacity  per unit weight of the adsorbent (mg/g),  C_e_ is the concentration of adsorbate at equilibrium (mg/ L)  and K_L_ is the Langmuir constant relating to the affinity of  the binding sites (L /mg). |
|  | $R_{L}=\frac{1}{1+K_{L}C_{0}}$ | The dimensionless constant $R_{L}$ determines the adsorption  type where $C_{0}$ is the initial concentration of  the adsorbate (mg/L). |
| Freundlich | $\ln q_{e}= \frac{1}{n_{f}}\ln C_{e}+\ln K_{f}$ | where K_f_ is the Freundlich constant relating to the sorption capacity (L/g ) and n_f_ is the sorption intensity of adsorbent (dimensionless).  These constants are obtained from plotting $\ln q_{e}$  against $\ln C_{e}$, resulting in a linear relationship |
| Temkin | $qe=B_{t}\ln A_{t} +B_{t}\ln C_{e}$ | Where A_t_ Temkin isotherm equilibrium binding  constant (L/g) b_t_ = Temkin isotherm constant  R= universal gas constant (8.314 J/mol/K)  T= Temperature at 298K.  B_t_ = Constant related to heat of sorption (J/mol) |
|  | B_t_=RT/b |  |

**Table S3: Thermodynamic models utilized to describe CV dye adsorption process onto LSR-F.**

| Model | Equation | Abbreviations |
| --- | --- | --- |
| Thermodynamic  (Arrhenius equation) | $\ln K_{D}= \ln A-(\frac{E_{a}}{RT})$ | Where 𝐸_𝑎_ is the activation energy (J/mol),  A is the Arrhenius constant (g/mg/min),  K_D_ is the pseudo-second-order rate  constant (g/mg/min), R is the ideal  gas constant (8.314 J/mol K),  and T is the temperature in Kelvin. |
| Thermodynamic parameters  (Van't Hoff equation) | $\ln K_{D} = \frac{-G◦}{\mathrm{RT}} = \frac{S◦}{R} - \frac{H◦}{\mathrm{RT}}$ | Here, 𝑅 is the gas constant (8.314 J/mol K),  𝑇 is the solution temperature in (K)  and 𝐾_𝐷_ is the adsorption equilibrium  constant (L/g). ∆𝐻^∘^, ∆𝑆^∘^, and 𝐺^∘^ represent  changes in enthalpy (Kj/mol), entropy (j/mol.K),  and gibbs free energy (Kj/mol), respectively. |
|  | $G◦= H◦-T S◦$ |  |


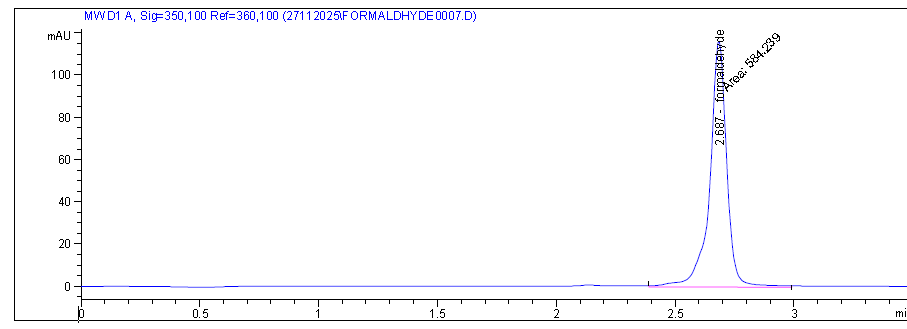


**pH = 2**


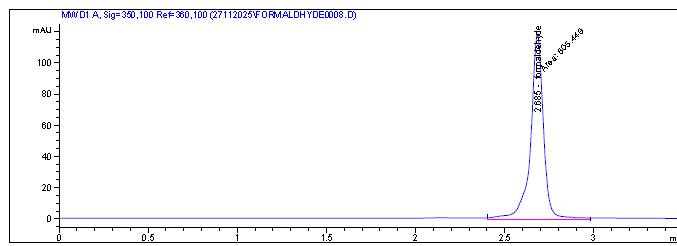


**pH = 4**


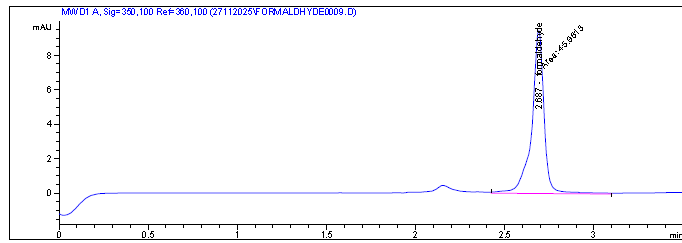


**pH = 6**


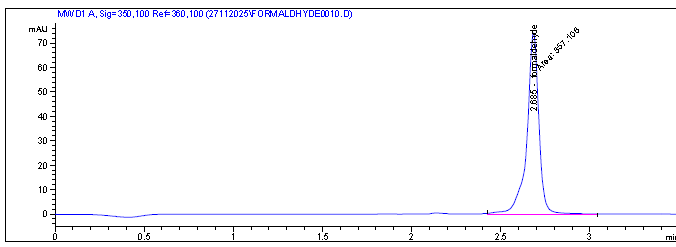


**pH =8**


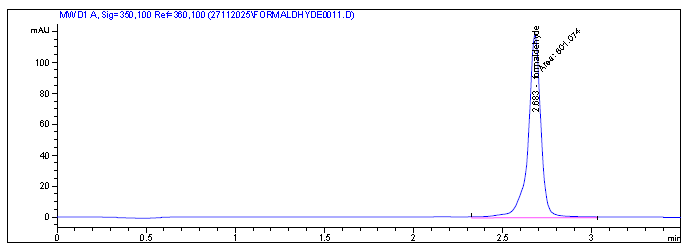


**pH = 10**


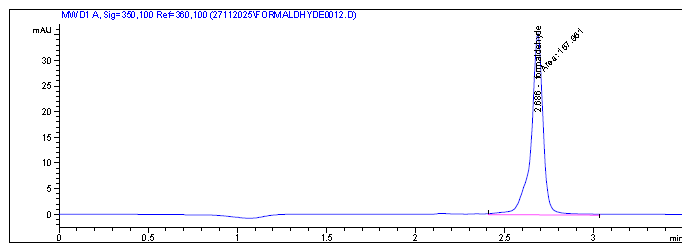


**pH = 12**

**Figure S1: HPLC for detection of formaldehyde in different pH (2-12).**

**References:**

1. Tan, H., Yang, R., Sun, W. & Wang, S. Peroxide - Acetic Acid Pretreatment To Remove Bagasse Lignin Prior to Enzymatic Hydrolysis. *Am. Chem. Soc.* **49**, 1473–1479 (2010).

2. Božnar, M. Z., Grašič, B., Oliveira, A. P. de, Soares, J. & Mlakar, P. Spatially transferable regional model for half-hourly values of diffuse solar radiation for general sky conditions based on perceptron artificial neural networks. *Renew. Energy* **103**, 794–810 (2017).

3. Racero-Galaraga, D., Rhenals-Julio, J. D., Sofan-German, S., Mendoza, J. M. & Bula-Silvera, A. Proximate analysis in biomass: Standards, applications and key characteristics. *Results Chem.* **12**, 101886 (2024).

4. Demirbas, A. Combustion characteristics of different biomass fuels. *Prog. Energy Combust. Sci.* **30**, 219–230 (2004).

5. Karam, F. F., Hassan, F. F. & Hessoon, H. M. Adsorption of toxic crystal violet dye using (Chitosan-OMWCNTs) from aqueous solution. *J. Phys. Conf. Ser.* **1999**, (2021).

6. Momina, Mohammad, S. & Suzylawati, I. Study of the adsorption/desorption of MB dye solution using bentonite adsorbent coating. *J. Water Process Eng.* **34**, (2020).
